# Effects of Crocus sativus on glycemic control and cardiometabolic parameters among patients with metabolic syndrome and related disorders: a systematic review and meta-analysis of randomized controlled trials

**DOI:** 10.1186/s12986-024-00806-y

**Published:** 2024-05-25

**Authors:** Xiaodan Yan, Shuyuan Zhao, Xue Feng, Xinrui Li, Qian Zhou, Qiu Chen

**Affiliations:** 1 Chengdu University of Traditional Chinese Medicine, Chengdu, 610032 China; 2 Hospital of Chengdu University of Traditional Chinese Medicine, Chengdu, 610032 China

**Keywords:** Crocus sativus, Crocin, Metabolic syndrome, Glycemic control, Cardiometabolic parameters

## Abstract

Metabolic syndrome (MetS) is a cluster of clinical syndromes that is closely associated with an elevated risk of developing atherosclerotic cardiovascular disease. In a series of animal experiments and clinical trials, crocus sativus and its component crocin have demonstrated promising hypoglycemic effects. However, there is currently insufficient evidence regarding their impact on cardiometabolic parameters. Our study aimed to assess the impact of Crocus sativus and crocin on glycemic control in individuals with metabolic syndrome and associated disorders, as well as their potential effects on improving cardiometabolic parameters. We searched Cochrane Library, PubMed, Embase, and Web of Science databases to ascertain the pertinent randomized controlled trials (RCTs) until December 30, 2023. Q-test and I^2^ statistics were utilized to evaluate heterogeneity among the included studies. Data were merged using a random-effects model and presented as (WMD) with a 95% confidence interval (CI). The current comprehensive review and meta-analysis, encompassing 13 RCTs involving a total of 840 patients diagnosed with metabolic syndrome and associated disorders, demonstrates that Crocus sativus was superior to placebo on Hemoglobin A1c(HbA1c) (WMD: -0.31;95% CI [-0.44,-0.19]. *P* = 0.002) and systolic blood pressure(SBP) (WMD:-7.49;95% CI [-11.67,-3.30]. *P* = 0.99) respectively. Moreover, Crocus sativus improved fasting blood glucose (FBG) (WMD:-7.25;95% CI [-11.82, -2.57]. *P* = 0.002) when used crocin and on other chronic diseases. Crocus sativus reduced the total cholesterol (TC) among the metabolic syndromepatients (WMD:-13.64;95%CI [-26.26, -1.03]. *P* = 0.03). We demonstrated that Crocus sativus exerts beneficial effects on glycemic control and cardiometabolic parameters in individuals with metabolic syndrome and related disorders.

**Supplementary Information:**

The online version contains supplementary material available at 10.1186/s12986-024-00806-y.

## Introduction

Metabolic syndrome is a medical disorder distinguished by a combination of metabolic risk factors, including high blood sugar levels, elevated blood pressure, abnormal lipid levels, uneven blood thickness, and excess body weight [1]. These risk factors can have adverse effects on blood vessels and endothelial cells, increasing the risk of atherosclerosis and other cardiovascular diseases [2]. This complaint has emerged as one of the top chronic ailments posing a global hazard to human health.

The high incidence of MetS and the high risk of cardiovascular diseases required more effective treatment methods. Traditional herbal therapy as a complementary approach may be an effective way to manage MetS [3]. The natural carotenoid compounds saffron, crocin, and crocetin exhibit anti-inflammatory, antioxidant, and neuroprotective properties.

Published research results have repeatedly confirmed that saffron can improve the increase of blood glucose and blood pressure caused by MetS and related diseases [4–6], protect vascular endothelial function, and even delay the process of diabetic nephropathy [7]. However, the effects of crocin on metabolic diseases vary in different clinical studies.

In preclinical studies, Algandaby, M. et al. discovered that crocin could inhibit the increase of blood glucose [8]. El-Fawal, R. et al. suggested that crocin can effectively regulate the levels of serum insulin, AGEs, TNF-α, and MDA, reverse the aortic damage and cardiac tissue structural changes induced by MetS, and protect the cardiovascular system [9].

Aynaz Tajaddini et al. revealed through clinical studies that safflower intervention can effectively reduce FBG, blood lipids, and liver enzymes, and improve oxidative status in type 2 diabetes mellitus (T2DM) [6]. However, Samaneh Sepahi et al. considered that there was no significant change in blood lipids and liver enzymes after crocin intervention in T2DM [10]. The differences in these results may be attributed to factors such as study design, characteristics of the population studied, and variations in saffron preparations, which influenced the heterogeneity of the experiments.

The aim of this research is to evaluate the effects of saffron and crocin on blood glucose control in MetS and related diseases, as well as their potential for improving cardiometabolic parameters.The investigation of heterogeneity was further enhanced through subgroup analyses, with a specific focus on the classification of chronic conditions.

## Methods

This article was composed in accordance with the guidelines of Preferred Reporting Items for Systematic Reviews and Meta-analyses (PRISMA) guidelines [11] The PRISMA checklist can be found in the Additional file 1. The protocol has been registered on the prospero platform (CRD42024500734).

### Search strategy and study selection

We searched the Cochrane Library, PubMed, Embase, and Web of Science databases until December 30, 2023, to identify the relevant RCTs that evaluate the effects of Crocus sativus L. (saffron) and crocin on glycemic control and cardiometabolic parameters among patients with metabolic syndrome and related disorders. We adopt the PICOS principle to formulate our search strategy. Patients: metabolic syndrome and related disorders (such as "metabolic disease," "MetS," "diabetes"); Intervention: (such as "saffron", "Crocus sativus"); Outcomes: (such as "FBG", "HbA1c"). A more detailed search strategy can be found in Additional file 2 .The electronic database search will be supplemented by a manual examination of the reference lists of the included articles. The search for publications was not restricted by date or language, ensuring comprehensive coverage. Two independent reviewers conducted the search and selection of randomized controlled trials (RCTs). Revisions were made based on the discussion with an additional reviewer to resolve any disagreements.

### Selection criteria

Trials with the following criteria were included in our meta-analysis:(1) Randomized clinical trials reported in full articles.(2) Patients aged 18 years or older with metabolic syndrome and related diseases. Metabolic syndrome and related diseases include metabolic syndrome, overweight, obesity, diabetes, polycystic ovary syndrome, hypertension, coronary heart disease, chronic kidney disease, non-alcoholic fatty liver disease, and hypercholesterolemia. (3) The interventions consist of oral saffron (saffron, saffron extract, crocin) or saffron combined with other interventions (referring to the application of basic treatment or standardized therapeutic measures in both the intervention and control groups). (4) At least one of the interest outcomes was reported.

### Exclusion criteria

We excluded animal experiments, in vitro studies, case reports and series, observational studies, trial protocols or abstracts without findings, as well as clinical trials lacking a control group.

### Data extraction

Two authors used prepared Excel worksheets to extract the data, and they consulted a third author if there were any differences. The following data will be extracted: (1) Risk of Bias assessment related data. (2) Characteristics of the studies, including study ID, study design, patient numbers, diagnosis, intervention and control groups, treatment duration, and protocol number. (3) Baseline data of patients, including disease duration, body mass index (BMI), and the number of participants in the intervention group, were collected. (4) Main outcomes data and additional outcomes data were collected, encompassing glycemic control parameters: FBG, HbA1c, fasting serum insulin (FINS), and homa-ir insulin resistance index (HOMA-IR), as well as cardiometabolic parameters: triglycerides (TG), TC, high density lipoprotein (HDL), low density lipoprotein (LDL), SBP, diastolic blood pressure(DBP), and BMI.

### Quality assessment

Two authors independently estimated the quality of the included RCTs using the Cochrane Collaboration risk of bias 2(ROB 2) tool [12]. This tool evaluates the quality of study methodology in various aspects, including the generation of sequences, concealment of allocation, blinding of participants, personnel, and outcome assessors, handling of incomplete outcome data, reporting of selective outcomes, and identification of other potential biases. All the studies included in this analysis were categorized into low, high, or uncertain risk of bias. We employ the Grade Tool (Grading of Recommendations, Assessment, Development, and Evaluation) for appraising the robustness of survey findings. The certainty of evidence is classified into three tiers: high, moderate, and low [13]. Any disagreements are resolved by the third author in a team discussion. The information was then summarized into a figure representing the risk of bias.

### Statistical analysis

The effect estimates for changes in glycemic control and cardiometabolic parameters for the intervention group and control groups were expressed as weighted mean differences (WMD) and 95% confidence intervals(95% CIs). In cases where the values were not explicitly stated in the studies, they were estimated. The detailed data synthesis strategies can be found in Supplementary Methods. The results will be presented using forest plots. The heterogeneity was evaluated by I^2^ statistic (No heterogeneity: I^2^ ≥ 0%; Mild heterogeneity: I^2^ ≥ 25%; Moderate heterogeneity: I^2^ ≥ 50%; Severe heterogeneity: I^2^ ≥ 75%). According to the Cochrane Handbook, we opted for a random effects model over a fixed effects model in consideration of the varied attributes identified within the studies that were incorporated, as a random effects model is better suited to account for the expected heterogeneity between studies [14]. All statistical analyses were conducted utilizing Review Manager 5.4.1 software (Nordic Cochrane Centre, The Cochrane Collaboration, Copenhagen, Denmark) and Stata version 17 (Stata Corp, College Station, TX, USA).

### Subgroup analysis

Subgroup analyses were performed to investigate the potential moderator variables that could account for the observed heterogeneity: different saffron preparations (Crocus sativus plant, extract vs crocin), duration of the intervention (≤ 8 vs > 8 weeks), different doses of crocin(≤ 50mg vs > 50mg), and types of chronic condition (MetS, T2DM vs other chronic diseases).

### Sensitivity analysis and publication bias

We performed sensitivity analyses for the main outcomes. Efforts were undertaken to evaluate the collective influence of specific research and subcategories on the main result. The Egger's regression test and funnel plot were employed to detect potential indications of publication bias among the trials included in this study. Statistical significance was determined for *P* values below 0.05.

## Result

### Results of the literature search and characteristics of the studies

**Fig. 1:**
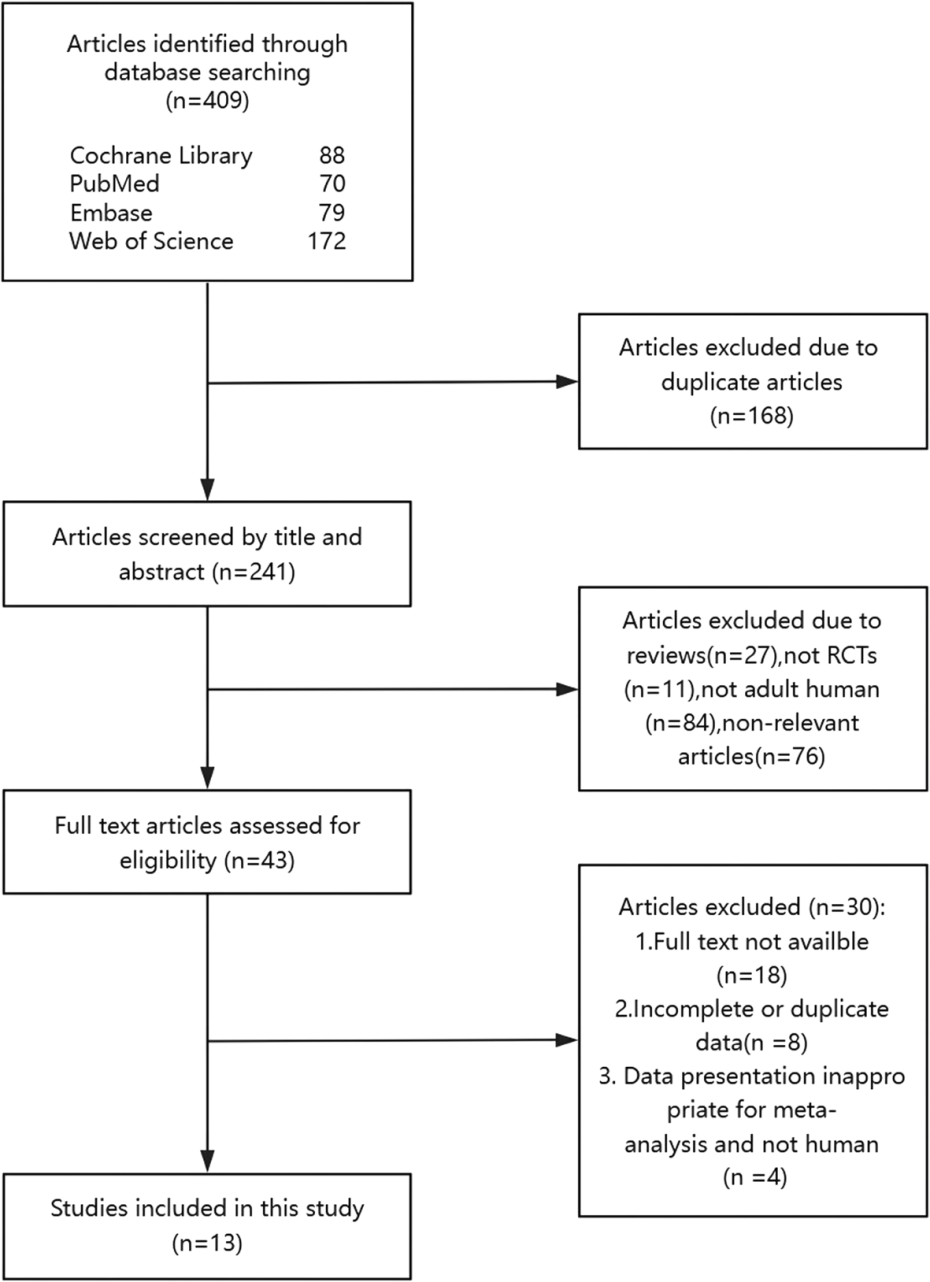
PRISMA flow diagram of study screening and selection

The process of search strategy results and study selection is illustrated in Fig. 1, providing a comprehensive overview. After a literature search without any restrictions, we found 407 records. After eliminating duplicate content and conducting a thorough examination of the selected research articles, we included 13 RCTs (or 15 effect sizes) [5–7, 10, 15–23] that satisfied the inclusion criteria. The study enrolled a total of 840 patients diagnosed with metabolic syndrome and related disorders, among whom 457 individuals were assigned to the intervention group and 383 individuals to the control group. The duration of the trials ranged from 6 weeks to 3 months, using different saffron preparations (saffron plants, saffron extracts, or crocin) with doses of crocin ranging from 15 mg to ~ 100 mg/day. In the included studies, patients retained their usual medication while intervening. The comprehensive attributes of the studies incorporated are presented in Table 1.

**Table 1:** Basic characteristics of included studies

Study ID	Study design	Patients (diagnosis, number)	Sample size(IN/CON)	Group IN	Group CON	Duration of treatment	Presented data	Age(y) (IN/CON)	BMI(kg/m^2^) (IN, CON)	Protocol number
Abedimanesh et al.(2017)(a) [15]	Triple arm study	Coronary heart disease (*n* = 37)	25/12	saffron aqueous extract(30mg/day)	placebo(30mg/day)	8 weeks	FBG,HbA1c,TC,TG,HDL,LDL,BMI	56.04 ± 7.55, 56.32 ± 5.91	28.64 ± 2.23, 28.05 ± 2.89	IRCT201512102017N26
Abedimanesh et al.(2017)(b) [15]	Triple arm study	Coronary heart disease (*n* = 38)	25/13	crocin(30mg/day)	placebo(30mg/day)	8 weeks	FBG,HbA1c,TC,TG,HDL,LDL,BMI	53.36 ± 5.94, 56.32 ± 5.91	27.92 ± 2.57, 28.05 ± 2.89	IRCT201512102017N26
Behrouz et al.(2020) [16]	Double arm study	T2DM (*n* = 50)	25/25	crocin(30mg/day)	placebo(30mg/day)	3 months	FBG,SBP,DBP,HbA1c,HOMA-IR,FINS	57.08 ± 7.41 59.86 ± 9.46	30.64 ± 4.79, 30.85 ± 3.19	NCT04163757
Ebrahimi et al.(2019) [17]	Double arm study	T2DM (*n* = 80)	40/40	saffron powder (100mg/day)	placebo powder(100mg/day)	12 weeks	SBP,DBP,FBG,HbA1c,FINS,HOMA-IR,TG,TC,HDL,LDL ,BMI	55.2 ± 7.3, 53 ± 10.6	29.3 ± 4.9, 30.5 ± 4.7	IRCT201510259472N9
Jaafarinia et al.(2022) [7]	Double arm study	Diabetic nephropathy (*n* = 40)	21/19	crocin(15mg/day)	placebo(15mg/day)	90 days	BMI,SBP,DBP,FBG,TC,TG,HDL,LDL,HbA1c,	63.8 ± 0.62, 62.68 ± 9.84	27.21 ± 3.86, 27.26 ± 3.34	IRCT20190810044500N4
Javandoost et al.(2017) [18]	Double arm study	MetS (*n* = 44)	22/22	crocin(60mg/day)	placebo(60mg/day)	8 weeks	FBG, TG, TC, HDL, LDL	NA	NA	IRCT2013080514279N1
Karimi-Nazari et al.(2019) [19]	Double arm study	Overweight/Obese prediabetes(*n* = 75)	36/39	saffron extract(pill,15mg/day)	placebo(pill,15 mg/day)	8 weeks	FBG,HbA1c,TC,TG,HDL,LDL,BMI	57.95 ± .12, 57.9 ± 8.7	29.35 ± 1.50, 28.78 ± 2.02	IRCT20120913010826N19
Kermani et al.(2017a) [20]	Double arm study	MetS (*n* = 48)	24/24	crocin(tablets,~100 mg/day)	placebo(tablets,~100 mg/day)	6 weeks	FBG, TC, TG, HDL,LDL,SBP,DBP,BMI	53.8 ± 9.2, 50.9 ± 8.8	29.9 ± 3.9, 29.8 ± 5.3	IRCT2016112617756N11
Kermani et al.(2017b) [21]	Double arm study	MetS (*n* = 44)	22/22	saffron(capsule,100 mg/day)	placebo(capsule,100 mg/day)	12 weeks	SBP,DBP,FBG,TC,TG,HDL,LDL	43.64 ± 11.1742.59 ± 8.44	31.02 ± 5.45, 30.48 ± 6.26	NA
Milajerdi et al.(2018) [22]	Double arm study	T2DM (*n* = 52)	26/26	saffron hydroalcoholic extracts(30mg/day)	placebo (30 mg/day)	8 weeks	FBG,HbA1c,TC,TG,HDL,LDL	54.57 ± 6.96, 55.42 ± 7.58	23.84 ± 11.89, 28.30 ± 3.24	IRCT2015082623776N1
Moravej et al.(2019) [5]	Double arm study	T2DM (*n* = 64)	32/32	hydroalcoholic extract of saffron(30mg/day)	placebo(30mg/day)	3 months	FBG,HOMA-IR,TG, TC, HDL, HbA1c	53.5 ± 9.9, 52.4 ± 13	28.8 ± 4.0, 27.5 ± 4.2	IRCT2015110219739N1
Nikbakht-Jam et al. (2016) [23]	Double arm study	MetS (*n* = 58)	29/29	crocin(30mg/day)	placebo(30mg/day)	8 weeks	FBG, TG, TC, HDL, LDL	38.97 ± 13.33 43.46 ± 12.77	NA	IRCT2013080514279N1
Sepahi et al.(2022)(a) [10]	Triple arm study	T2DM (*n* = 75)	50/25	crocin(30mg/day)	placebo(30mg/day)	3 months	FBG,HbA1c,FINS,HOMA-IR, TC,TG,HDL,LDL	57.58 ± 1.0, 56.92 ± 1.9	NA	IRCT2015101713058N3
Sepahi et al.(2022)(b) [10]	Triple arm study	T2DM (*n* = 75)	50/25	saffron extract(30mg/day)	placebo(30 mg/day)	3 months	FBG,HbA1c,FINS,HOMA-IR, TC,TG,HDL,LDL	57.16 ± 1.5, 56.92 ± 1.9	NA	IRCT2015101713058N3
Tajaddini et al.(2023) [6]	Double arm study	T2DM (*n* = 60)	30/30	saffron powder (100mg/day)	placebo powder(100mg/day)	8 weeks	FBG,HbA1c,HOMA-IR,TC,TG,HDL,LDL,BMI	50.57 ± 9.88, 51.83 ± 10.91	30.0 ± 4.2, 31.2 ± 4.6	IRCT20090609002017N24

### Risk of bias and Grade assessment

**Fig. 2:**
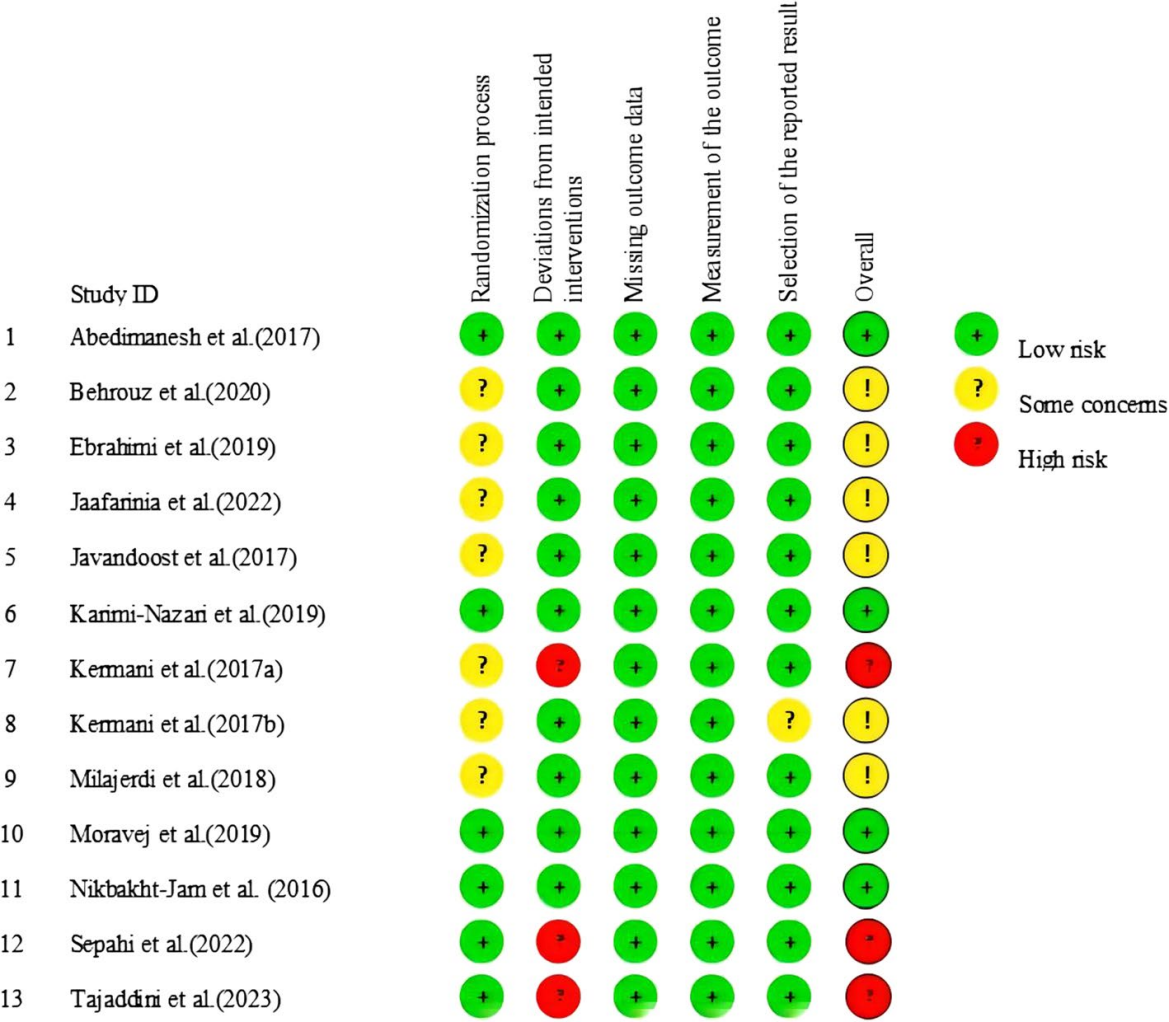
The methodological quality of included studies (risk of bias)

Among the 13 studies included in the meta-analysis, four had a low risk of bias, six demonstrated some concern, while the other three showed a high risk of bias. In seven studies, there was a lack of detail in describing the stages of the randomization process, which raises some concerns; one of these is also unclear in the selection of the reported result. Three studies with a high risk of bias resulted from the question of deviations from the intended intervention. The comprehensive summary of the risk of bias assessment is presented in Fig. 2. According to the grade-based evaluation, there is a high level of certainty in the evidence for HbA1c, SBP, DBP, and BMI. The evidence for FBG, TC, HDL, and LDL has a moderate level of certainty. However, the evidence for FINS, HOMA-IR, and TG has a low level of certainty. Additional file 3 provides detailed descriptions of the grading system used for each outcome measure.

### Pooled analysis of all studies

#### Pooled analysis of glycemic control parameters

**Fig. 3:**
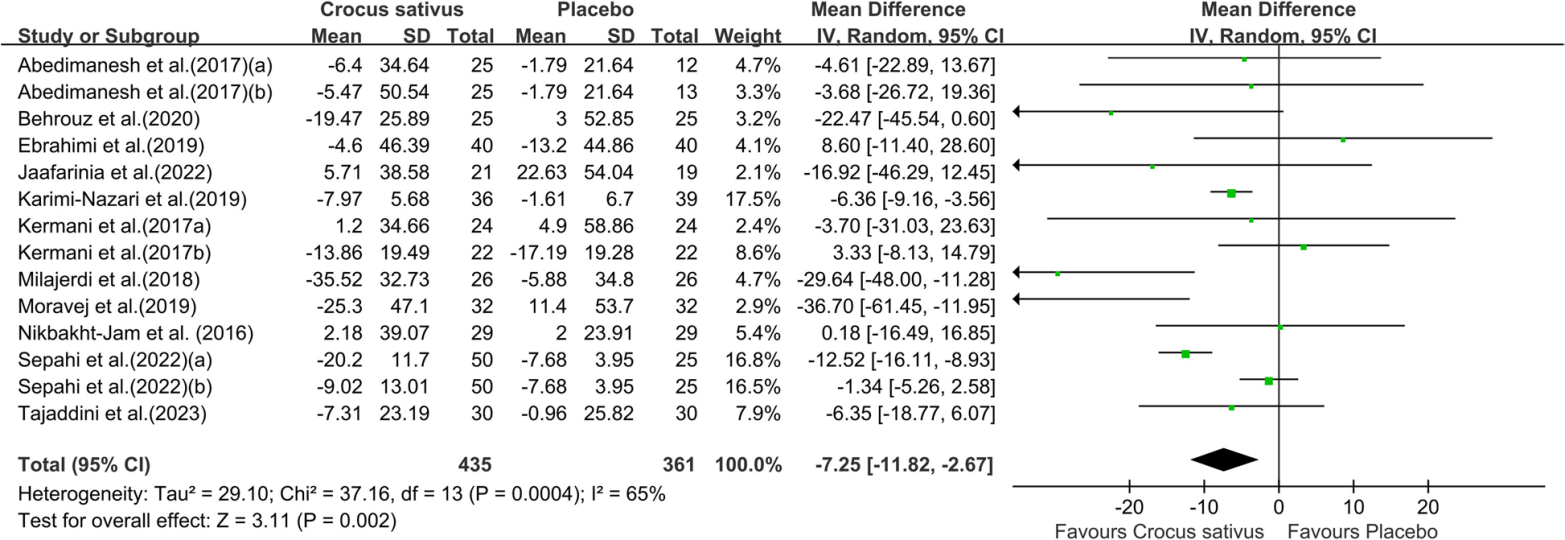
Forest plot of the efficacy of Crocus sativus on FBG

**Fig. 4:**
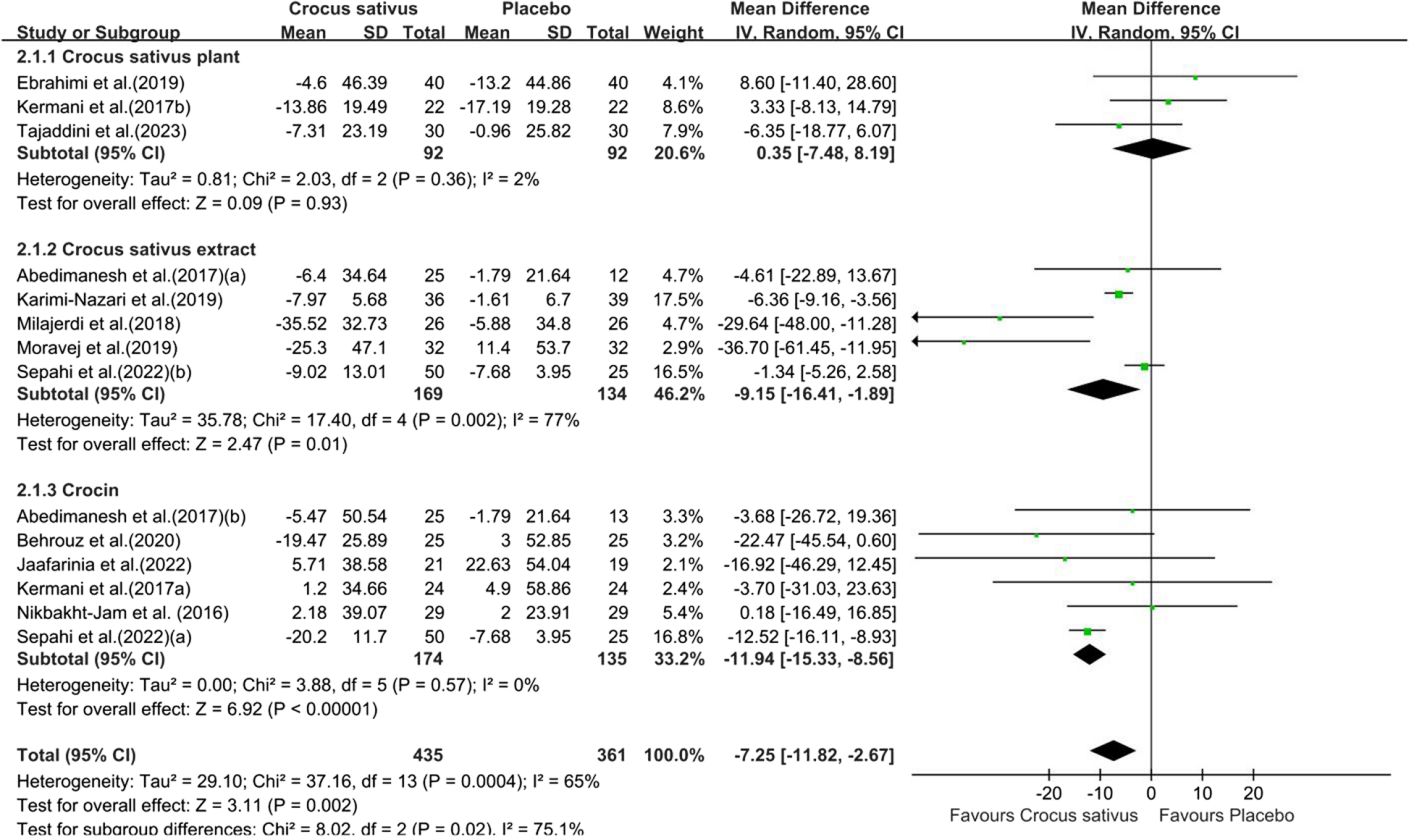
Forest plot of subgroup analysis by saffron preparations of the estimated impact of Crocus sativus on FBG

**Fig. 5:**
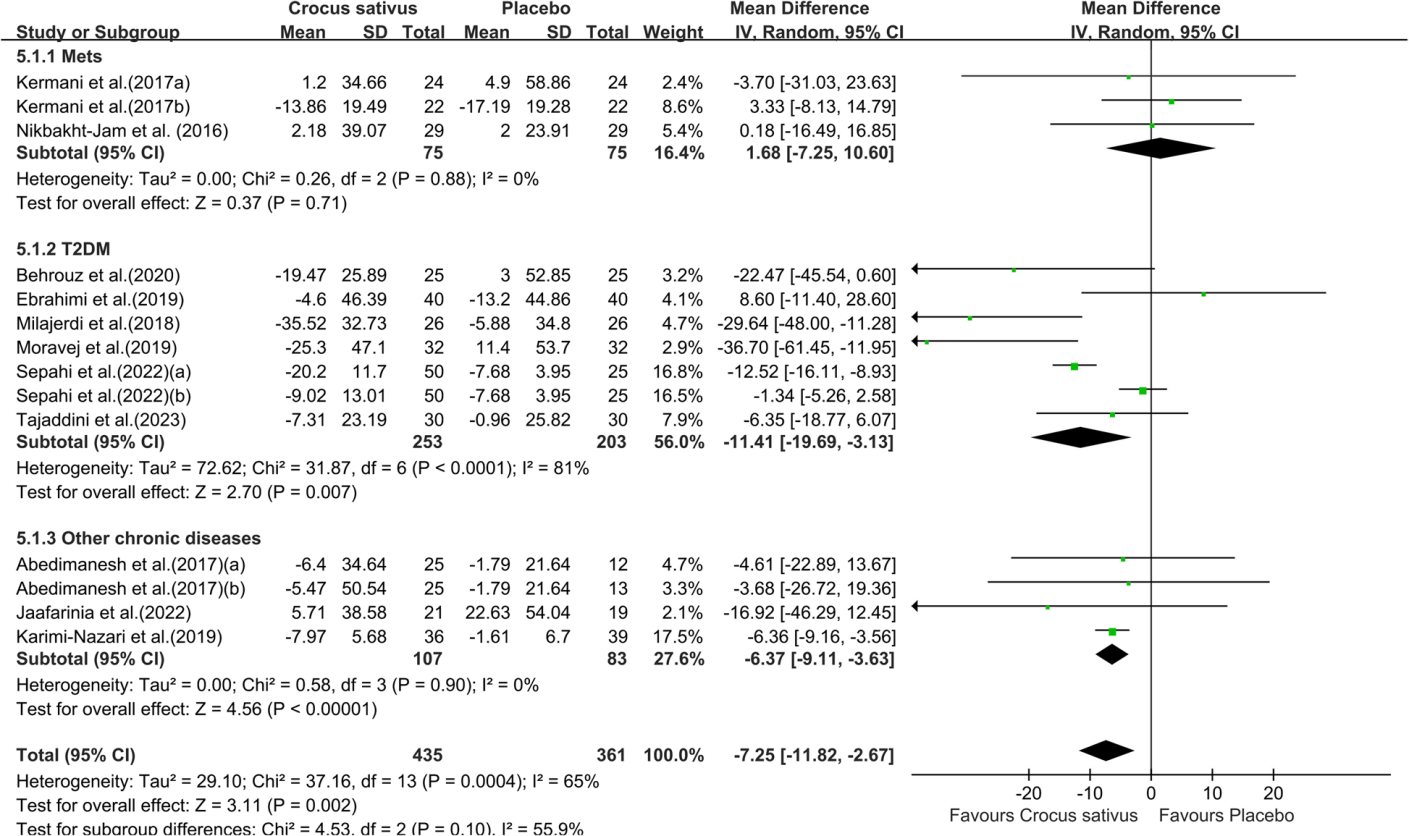
Forest plot of subgroup analysis by type of chronic condition of the estimated impact of Crocus sativus on FBG

Combining twelve studies with fourteen effect sizes, we reached that the group receiving the intervention had a significant reduction in FBG in comparison to the control group (WMD: -7.25;95% CI [-11.82, -2.67]. *P* = 0.002, Fig. 3). However, due to the moderate between-study heterogeneity (I^2^ = 65%), we performed subgroup analysis. The results showed that saffron preparations and duration of intervention type of chronic condition may be the source of heterogeneity. Crocus sativus significantly reduced FBG within studies conducted that used crocin (WMD:-7.25;95% CI [-11.82,-2.57]. *P* = 0.002, Fig. 4), duration of intervention (≤ 8 weeks)(WMD:-6.97;95% CI [-11.50,-2.43]. *P* = 0.003, Fig. S1a) and on other chronic diseases (WMD:-6.37;95% CI [-9.11,-3.63]. *P* < 0.00001, Fig. 5).The pooled studies show low heterogeneity in above subgroups (I^2^ = 0%,13%,0%). Nevertheless, the effect was indistinctive among other subgroups (Fig. S1b).

**Fig. 6:**
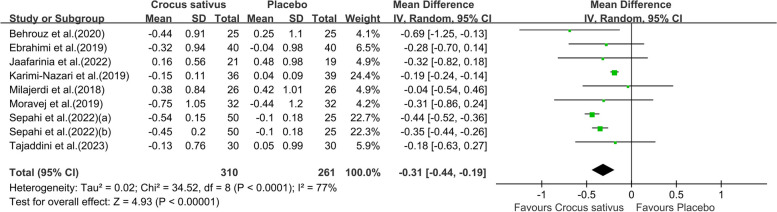
Forest plot of the efficacy of Crocus sativus on HbA1c

**Fig. 7:**
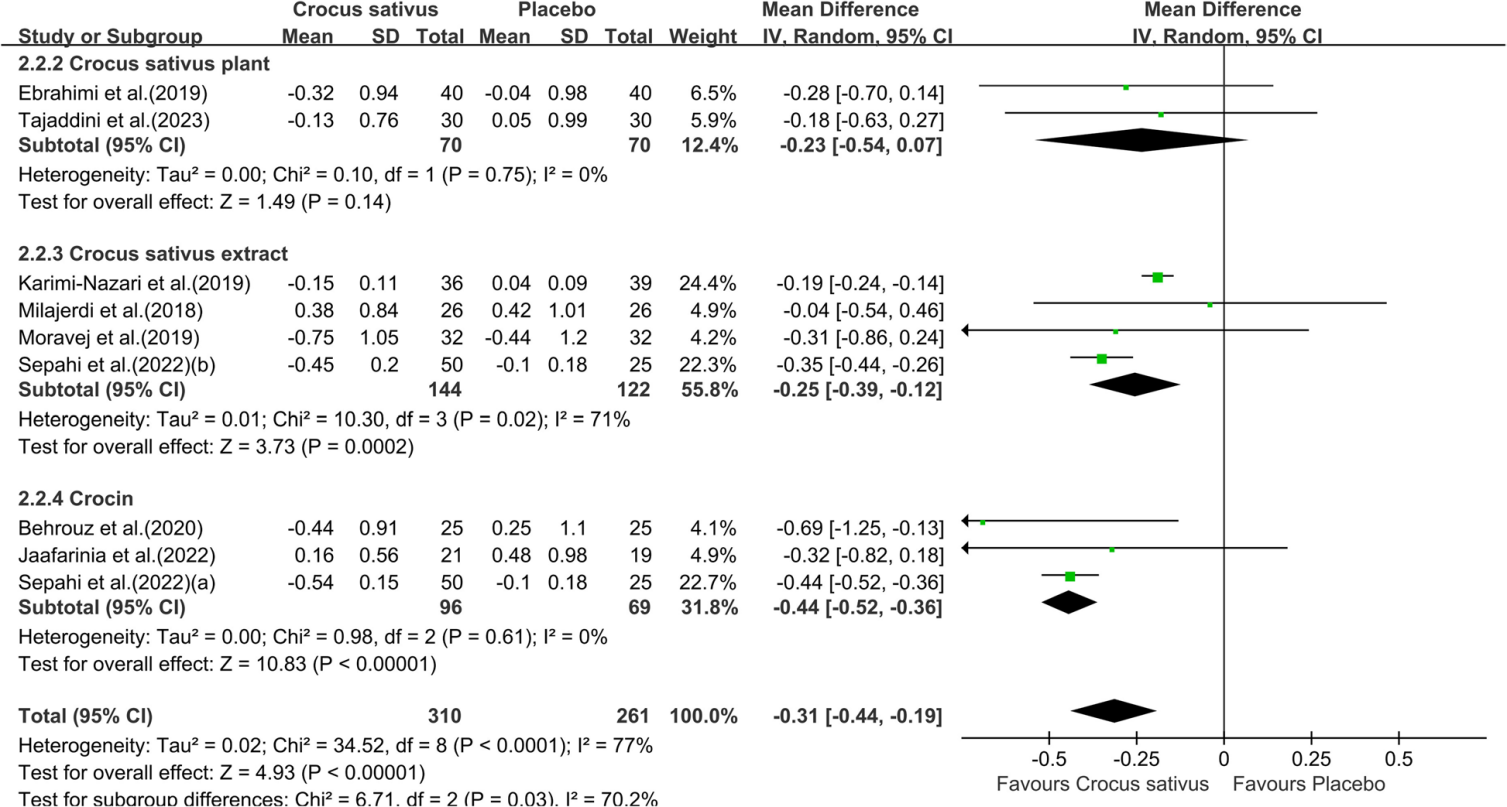
Forest plot of subgroup analysis by saffron preparations of the estimated impact of Crocus sativus on HbA1c

**Fig. 8:**
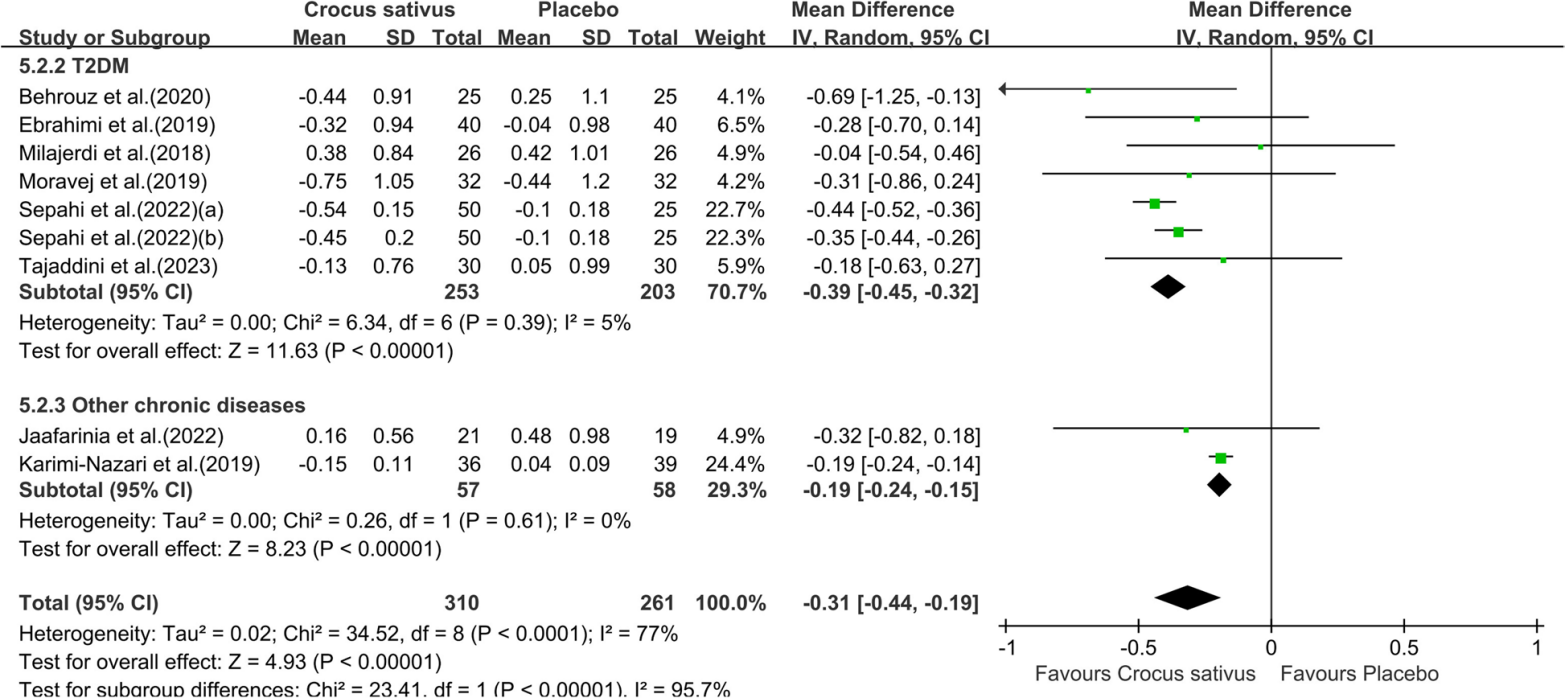
Forest plot of subgroup analysis by type of chronic condition of the estimated impact of Crocus sativus on HbA1c

Pooling effect sizes from eight studies with nine effect sizes, the effect of Crocus sativus on HbA1c was significant (WMD:-0.31;95% CI [-0.44, -0.19]. *P* = 0.002, Fig. 6). The results showed high heterogeneity (I^2^ = 77%). When the study by Karimi-Nazari et al was removed [19], the heterogeneity of study results on HbA1c became insignificant (I^2^ = 0%). We infer that the study leads to heterogeneity across studies. The impact of Crocus sativus on HbA1c remained unchanged (WMD:-0.39;95% CI [-0.45, -0.33]. *P* < 0.00001), thus suggesting that study quality did not influence this result. Subgroups analysis showed crocin was more effective (WMD:-0.44;95% CI [-0.52,-0.36]. *P* < 0.00001, Fig. 7)in reducing HbA1c than Crocus sativus plant and extract with less heterogeneity (I^2^ = 0%) and Crocus sativus has a better effect (WMD:-0.39;95% CI [-0.45,-0.33]. *P* < 0.00001, Fig. 8) on improving HbA1c in T2DM patients with little heterogeneity (I^2^ = 5%).Additional subgroup results are presented in the Supplementary data (Fig. S2).

Pooling the effect sizes of the five studies, the effect of Crocus sativus on fasting serum insulin concentration was not significant (WMD: -0.68;95% CI [-1.27,-0.34]. *P* = 0.54). Similarly, no significantly different effects were observed for HOMA-IR (WMD:0.07;95% CI [-0.8,0.94]. *P* = 0.88). In subgroup analysis, although there is significant heterogeneity among subgroups, they cannot eliminate heterogeneity (Fig. S3a-c, S4a-c).

### Pooled analysis of cardiometabolic parameters

A total of ten publications with twelve effect sizes reported TG concentrations. Crocus sativus had no statistically significant effect on TG (WMD: -5.15;95% CI [-10.81,0.51]. *P* = 0.08, Fig.S5) with mild heterogeneity (I^2^ = 39%). Subgroup analysis reached the same conclusion, and none of the factors significantly influenced the results.

The analysis of TC included a total of twelve publications with fourteen effect sizes. There were no notable differences observed in the intervention group when compared to the control group (WMD: -4.44;95% CI [-9.71,0.83]. *P* = 0.1, Fig.S6). There was a moderate level of heterogeneity observed among the studies (I^2^ = 34%). The subgroup analysis, which was categorized based on disease type, is presented in Table 2. Another subgroup analysis was concluded as not statistically significant.

**Table 2:** Subgroup analysis to assess the effect of Crocus sativus on different types of disease

Main outcome measures	Disease types	No of trials	Sample size(IN,CON)	Effect size	95% CI	I-squared (%)	P for heterogeneity	I-squared between subgroup (%)	P for between subgroup heterogeneity
FBG	MetS	3	75,75	1.68	-7.25,10.60	0	0.71	55.9	1
	T2DM	7	253,203	-11.41	-19.69,-3.13	81	0.007		
	Others	4	107,83	-6.37	-9.11,-3.63	0	< 0.0001		
HbA1c	MetS	NA	NA	NA	NA	NA	NA	95.7	< 0.0001
	T2DM	7	253,203	-0.39	-0.45, -0.32	5	< 0.0001		
	Others	2	57,58	-0.19	-0.24, -0.15	0	< 0.0001		
TG	MetS	2	46,46	-9.83	-39.05,19.39	0	0.51	0	0.92
	T2DM	6	228,178	-6.35	-14.47,1.77	39	0.13		
	Others	4	107,83	-10.38	-30.36,9.60	50	0.31		
TC	MetS	4	97,97	-13.64	-26.26,-1.03	0	0.03	61	0.08
	T2DM	6	228,178	-5.61	-14.17,2.95	44	0.11		
	Others	4	432,358	-0.17	-3.32,2.97	0	0.52		
HDL	MetS	3	75,75	-0.39	-3.43,2.65	0	0.8	0	0.72
	T2DM	6	228,178	0.01	-1.78,1.80	7	0.99		
	Others	4	107,83	0.73	-0.63,2.10	0	0.29		
LDL	MetS	3	75,75	-7.5	-28.35,15.35	60	0.48	17.3	0.30
	T2DM	6	228,178	-4.95	-13.73,3.83	51	0.27		
	Others	4	107,83	1.38	-1.38,4.14	0	0.33		
SBP	MetS	1	24,24	-3.30	-11.42,4.82	0	0.43	0	0.5
	T2DM	2	65,65	-8.92	-13.97,-3.86	0	0.0006		
	Others	1	21,19	-10.05	-28.44,8.34	0	0.28		
DBP	MetS	1	24,24	0.7	-8.55,9.95	0	0.88	0	0.93
	T2DM	2	65,65	0.24	-3.86,4.35	0	0.91		
	Others	1	21,19	-1.45	-9.65,6.75	0	0.73		
BMI	MetS	1	24,24	0.7	-1.82,3.22	0	0.54	0	0.46
	T2DM	2	70,70	0.27	-0.61,1.15	0	0.55		
	Others	4	107,83	-0.26	-0.71,0.18	0	0.25		

The impact of Crocus sativus on HDL levels did not reach statistical significance based on eleven publications with thirteen effect sizes (WMD:0.37;95% CI [-0.65,1.39]. *P* = 0.08, Fig. S7) and the heterogeneity is little (I^2^ = 0%). LDL was assessed in eleven studies with thirteen effect sizes. There was no statistical significance (WMD: -3.41;95% CI [-9.01,2.18]. *P* = 0.08, Fig.S8) with mild heterogeneity between the studies (I^2^ = 46%). All subgroup analyses were not statistically significant.

**Fig. 9:**
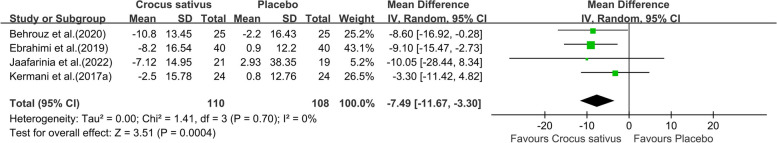
Forest plot of the efficacy of Crocus sativus on SBP

SBP was reported in four studies involving 218 patients. Overall statistical analyses concluded Crocus sativus significantly reduced systolic blood pressure levels compared with placebo (WMD: -7.49;95% CI [-11.67,-3.30]. *P* = 0.99, Fig. 9). In contrast, Crocus sativus on DBP showed no statistical significance among the same four studies (WMD:0.01;95% CI[-3.40,3.42]. *P* = 0.08, Fig. S9). No evidence of publication bias was found in the assessment of the effects of Crocus sativus (I^2^ = 0%). All subgroup factors did not affect the results.

A total of six studies with seven effect sizes measured BMI. General statistical analyses showed Crocus sativus was not superior to controls in reducing BMI (WMD:-0.13;95% CI [-0.53,0.26]. *P* = 0.51, Fig. S10), and there was low heterogeneity (I^2^ = 0%). Subgroup analyses did not show different results.

### Subgroup analysis and Sensitivity analysis

The impact of Crocus sativus on different types of diseases (MetS, T2DM, and other chronic diseases) was evaluated through a comprehensive subgroup analysis. A thorough examination was conducted for this purpose. The findings of the subgroup analysis are presented in Table 2. The funnel plot and Egger’s regression were used to assess publication bias. No indications of publication bias were detected in the evaluation of the impacts of Crocus sativus on FBG(*P* = 0.892), TG(*P* = 0.394), TC(*P* = 0.697), HDL(*P* = 0.094), LDL(*P* = 0.831). Visual funnel plots (Fig. S11a-k) showed no significant bias for all indicators. Our sensitivity analysis using stata 17 showed that Crocus sativus had a stable effect on improving FBG levels (Fig. S12).

## Discussion

### Summary of results and Comparison to previous studies

To the best of our understanding, this research represents the initial meta-analysis that examines the impact of saffron on glycemic control and cardiometabolic parameters in individuals with metabolic syndrome and associated conditions.

According to the results derived from this analysis, we found that: (1) Crocin significantly improves FBG compared with placebo and this result is more stable than that of Crocus sativus plant and extract. (2) Crocus sativus significantly reduces HbA1c and SBP levels. Moreover, intervention duration longer than 8 weeks was more effective in reducing HbA1c. (3) Among patients with MetS, Crocus sativus reduced TC levels.

It is postulated that the reduction in FBG and HbA1c observed with crocin may be attributed to its anti-inflammatory and antioxidant properties, specifically through scavenging ROS [24, 25]. Furthermore, another study has proposed that the anti-diabetic effects of Crocus sativus and its extracts may be associated with the promotion of regeneration of pancreatic beta-cells [26].

Research findings indicate that the utilization of Crocus sativus has been associated with a potential decrease in overall cholesterol levels by the inhibition of lipid synthesis and facilitation of macrophage polarization, thereby suppressing lipogenesis and regulating cholesterol homeostasis through downregulation of SREBP-1 [27, 28]. This measure would reduce the likelihood of atherosclerosis occurrence among individuals diagnosed with metabolic syndrome.

In recent years, the application of saffron in the fields of medicine has witnessed a significant surge in healthcare, agriculture, and cosmetics. Due to an imbalance between demand and supply, its price has consistently surged. Numerous studies have demonstrated that saffron encompasses a diverse range of active constituents such as carotenoids, flavonoids, terpenes, amino acids, and alkaloids [29, 30]. The primary mode of action for these active ingredients involves the inhibition of inflammatory responses and scavenging of reactive oxygen species, as well as the upregulation of sirtuin 1 (SIRT1) and nuclear factor erythroid 2-related factor 2 (Nrf2) expression. Additionally, they downregulate the nuclear factor kappa B (NF-κB) signaling pathway while suppressing inducible nitric oxide synthase and cyclooxygenase-2 (COX2), ultimately leading to improved organ dysfunction [31]. Thus, it accomplishes functions such as alleviating anxiety, reducing inflammation, providing antioxidant effects, combating viral infections and cancerous growths, regulating blood sugar and lipid levels, as well as enhancing memory [29, 32, 33].

The research findings suggest that saffron is extensively employed in the management of cardiovascular diseases, mental disorders, neurodegenerative diseases, atherosclerosis, cognitive impairments including learning and memory deficits, depression, diabetes, and cancer [34–38] due to its minimal toxicity and remarkable therapeutic efficacy [30, 39]. However, the efficacy of saffron in managing metabolic syndrome remains inconclusive. This study aims to evaluate the impact of saffron on blood glucose regulation and cardiac metabolism parameters in individuals with metabolic syndrome and related disorders, thereby substantiating its effectiveness in managing this condition. These findings will enhance clinical perspectives, augment both clinical and economic value, and necessitate further research and promotion.

Contrary to the results of a previous review that suggested saffron does not improve cardiovascular risk factors [40], we suggest that Crocus sativus has some control of blood glucose and lipids. The meta-analysis by Tahmasbi et al [41] concluded that saffron had no significant effect on HbA1c in overweight patients, which is different from our conclusion that Crocus sativus could improve HbA1c, while only Crocin had a significant FBG lowering effect.

Previous research analyzed the blood pressure-lowering effects of Crocus sativus in a review, but due to its small overall effect size (reducing SBP by 0.65mmHg and DBP by 1.23mmHg), it may not reach clinical importance [42]. Our study suggested that Crocus sativus had a significant lowering effect on SBP but had no effect on DBP. Compared with previous studies, our research suggests that Crocus sativus has a more significant effect in reducing blood pressure in patients with Mets and related diseases, which may have clinical significance. Since only four RCTs were included, the blood pressure-lowering effect of Crocus sativus needs to be further studied. A recent review showed that different saffron preparations have hypoglycemic activity in T2DM patients, and crocin may be more effective than other preparations [43].

### Strengths and limitations

We included RCT studies in the last decade. Besides investigating the hypoglycemic effect of Crocus sativus, this is the first meta-analysis of Crocus sativus on cardiometabolic parameters in patients with metabolic syndrome, which has some clinical implications.

Some of the studies had small sample sizes, and although subgroup analyses were performed, there may have been differences in the specific methods of implementation between the different interventions. Some clinical studies on Crocus sativus intervention in metabolic syndrome-related diseases were not included in this review due to a lack of data and non-normally distributed data presented in the form of median and interquartile range. Although most of the studies were at low or medium risk of bias, our meta-analysis of different outcome measures still showed some heterogeneity, ranging from low to moderate, which may be related to the intervention, study disease, population, ethnic region, and other factors. There are three pieces of evidence with low certainty for the outcome indicators, along with an additional four moderate-certainty ones.Therefore, we need to include more comprehensive, high-quality, and large-sample RCTS to further confirm the relevant conclusions.

## Conclusion

In conclusion, oral administration of Crocus sativus demonstrates a beneficial impact on FBG, HbA1c, and SBP in patients with metabolic syndrome and associated disorders. Additionally, Crocus sativus reduced TC level in patients with metabolic syndrome. Therefore, Crocus sativus may potentially contribute to the amelioration of metabolic syndrome and mitigation of cardiovascular events by serving as an agent for glycemic control and modulation of cardiometabolic parameters.

### Supplementary Information

Additional file 1. The PRISMA checklist

Additional file 2.  Methods for Systematic Review

Additional file 3. Results of the grading assessment for each outcome

Additional file 4: FigureS1a, FigureS1b, FigureS2, FigureS3a, FigureS3b, FigureS3c, FigureS4a, FigureS4b, FigureS4c, FigureS5- FigureS10. Forest plot of subgroup analysis by duration of intervention of the estimated impact of Crocus sativus on FBG. Forest plot of subgroup analysis by a dose of crocin of the estimated impact of Crocus sativus on FBG. Forest plot of subgroup analysis by duration of intervention of the estimated impact of Crocus sativus on HbA1c. Forest plot of the efficacy of Crocus sativus on FINS. Forest plot of subgroup analysis by saffron preparations of the estimated impact of Crocus sativus on FINS. Forest plot of subgroup analysis by duration of intervention of the estimated impact of Crocus sativus on FINS. Forest plot of the efficacy of Crocus sativus on HOMA-IR. Forest plot of subgroup analysis by saffron preparations of the estimated impact of Crocus sativus on HOMA-IR. Forest plot of subgroup analysis by duration of intervention of the estimated impact of Crocus sativus on HOMA-IR. Forest plot of the efficacy of Crocus sativus on TG. Forest plot of the efficacy of Crocus sativus on TC. Forest plot of subgroup analysis by type of chronic condition of the estimated impact of Crocus sativus on HDL. Forest plot of the efficacy of Crocus sativus on LDL. Forest plot of the efficacy of Crocus sativus on DBP. Forest plot of the efficacy of Crocus sativus on BMI. 

Additional file 5: Figure S11a-Figure S11k. Funnel plot of FBG. Funnel plot of HbA1c. Funnel plot of FINS. Funnel plot of HOMA-IR. Funnel plot of TG. Funnel plot of TC. Funnel plot of  HDL. Funnel plot of LDL. Funnel plot of SBP. Funnel plot of DBP. Funnel plot of BMI.

Additional file 6: Figure S12a, Figure S12b, Figure S12c. Sensitivity analysis of FBG. Sensitivity analysis of HbA1c. Sensitivity analysis of SBP.

## Data Availability

No datasets were generated or analysed during the current study.

## Abbreviations

BMI: Body mass index
CON: Control group
DBP: Diastolic blood pressure
FBG: Fasting blood glucose
FINS: Fasting insulin
HbA1c: Hemoglobin A1c
HDL: High-density lipoprotein
HOMA-IR: Homa-ir insulin resistance index
IN: Intervention group
LDL: Low-density lipoprotein
MetS: Metabolic syndrome
RCT: Randomized controlled trial
SBP: Systolic blood pressure
TC: Total cholesterol
TG: Triglycerides
T2DM: Type 2 Diabetes Mellitus
WMD: Weighted mean difference
