# Supplementary material for: Effects of Crocus sativus on glycemic control and cardiometabolic parameters among patients with metabolic syndrome and related disorders: a systematic review and meta-analysis of randomized controlled trials
Source: Nutr Metab (Lond). 2024 May 25;21:28. doi: 10.1186/s12986-024-00806-y (PMC11127410; doi:10.1186/s12986-024-00806-y)
Supplement: Supplementary file 2 — Additional file 2. Methods for Systematic Review [file 12986_2024_806_MOESM2_ESM.docx]

**Methods for** **Systematic Review**

**Search strategy**

The following MeSH and text keywords were used: patients("metabolic disease" OR "MetS" OR "diabetes" OR "T1DM" OR "T2DM" OR "overweight" OR "obese" OR "polycystic ovary syndrome" OR "hypertension" OR "blood pressure" OR "coronary heart disease" OR "chronic kidney disease" OR "nonalcoholic fatty liver disease" OR "hypercholesterolemia" ), AND intervention ("Saffron" OR "Saffrons" OR "Crocus sativus L." OR "Crocus sativus" OR "Crocin" OR "Crocus autumnalis Sm." OR "Crocus orsinii Parl." OR "Crocus pendulus Stokes" OR "Crocus sativus var. cashmerianus Royle" OR "Crocus sativus var. officinalis L." OR "Crocus setifolius Stokes" OR "Safran officinarum Medik"),AND outcomes("fasting blood glucose" OR "FBG" OR "HbA1c" OR “fasting serum insulin" OR "homeostasis model assessment of insulin resistance" OR "HOMAIR"OR "triglycerides" OR " TG" OR "total cholesterol" OR "TC" OR "LDL cholesterol" OR "LDL-C" OR "HDL-cholesterol" OR "HDL-C" OR "systolic blood pressure" OR "diastolic blood pressure" OR "inflammatory factors" OR "Inflammatory marker" OR "BMI" OR "Body Mass Index").

**Data synthesis strategy**

We extracted the mean, standard deviation (SD) for baseline and posttreatment from original publications. We calculated the mean and SD of the change from baseline according to the following equation from the Cochrane handbook.

·$mean difference=mean_{post-treatment}-mean_{baseline}$

·$\text{SD}_{\text{change}}=\sqrt{SD^{2}_{post-treatment}+SD^{2}_{baseline}-(2\times C\text{orr}\times SD_{post-treatment}\times SD_{\text{b}aseline})}$

The correlation coefficients (Corr) for FPG,HbA1c,TG,TC,HDL,LDL,SBP,DBP,and BMI were calculated according to the following equation using data from Jaafarinia et al. [1] as this study reported means and SD for change as well as for baseline and post-treatment. The correlation coefficients (Corr)for FINS and HOMA-IR was presumed to be 0.5.

·$Corr=\frac{{SD}_{baseline}^{2}+{SD}_{post-treatment}^{2}-{SD}_{change}^{2}}{2\times{SD}_{baseline}\times{SD}_{post-treatment}}$

For studies that reported medians and interquartile ranges (IQR), we estimated the mean and SD using an online calculator (<https://www.math.hkbu.edu.hk/~tongt/papers/median2mean.html>) as described in Luo, et al. [2] and Wan, et al. [3]. However, when the data were not normally distributed, we eliminated the data rather than transformed it.

**References**

1. Jaafarinia A, Kafami B, Sahebnasagh A, Saghafi F. Evaluation of therapeutic effects of crocin in attenuating the progression of diabetic nephropathy: a preliminary randomized triple-blind placebo-controlled trial. BMC complementary medicine and therapies. 2022;22(1):262. doi: 10.1186/s12906-022-03744-5.

2. Luo D, Wan X, Liu J, Tong T. Optimally estimating the sample mean from the sample size, median, mid-range, and/or mid-quartile range. Stat Methods Med Res. 2018;27(6):1785-805. doi: 10.1177/0962280216669183.

3. Wan X, Wang W, Liu J, Tong T. Estimating the sample mean and standard deviation from the sample size, median, range and/or interquartile range. BMC Med Res Methodol. 2014;14:135. doi: 10.1186/1471-2288-14-135.
