# Supplementary material for: Effects of Crocus sativus on glycemic control and cardiometabolic parameters among patients with metabolic syndrome and related disorders: a systematic review and meta-analysis of randomized controlled trials
Source: Nutr Metab (Lond). 2024 May 25;21:28. doi: 10.1186/s12986-024-00806-y (PMC11127410; doi:10.1186/s12986-024-00806-y)
Supplement: Supplementary file 3 — Additional file 3. Results of the grading assessment for each outcome [file 12986_2024_806_MOESM3_ESM.docx]

**Supplmentary Table 1**

Results of the grading assessment for each outcome

|  | **Certainty assessment** | | | | | | | **№ of patients** | | **Effect** | **Certainty** |
| --- | --- | --- | --- | --- | --- | --- | --- | --- | --- | --- | --- |
| **Outcomes** | **№ of studies** | **Study design** | **Risk of bias** | **Inconsistency** | **Indirectness** | **Imprecision** | **Other considerations** | **Pooled analysis of all studies** | **placebo** | **Absolute**  **(95% CI)** |  |
| FBG | 14 | randomised trials | not serious | not serious | not serious | serious^a^ | none | 435 | 361 | MD **7.25 lower**  (11.82 lower to 2.67 lower) | ⨁⨁⨁◯  Moderate |
| HbA1c | 9 | randomised trials | not serious | serious^b^ | not serious | not serious | strong association | 310 | 261 | MD **0.31 lower**  (0.44 lower to 0.19 lower) | ⨁⨁⨁⨁  High |
| FINS | 5 | randomised trials | serious^c^ | serious^b^ | not serious | not serious | none | 195 | 145 | MD **0.68 higher**  (1.47 lower to 2.84 higher) | ⨁⨁◯◯  Low |
| HOMA-IR | 6 | randomised trials | serious^d^ | serious^b^ | not serious | not serious | none | 227 | 177 | MD **0.07 higher**  (0.8 lower to 0.94 higher) | ⨁⨁◯◯  Low |
| TG | 12 | randomised trials | serious | not serious | not serious | serious^a^ | none | 381 | 307 | MD **5.15 lower**  (10.81 lower to 0.51 higher) | ⨁⨁◯◯  Low |
| TC | 14 | randomised trials | not serious | not serious | not serious | serious^a^ | none | 432 | 358 | MD **4.44 lower**  (9.71 lower to 0.83 higher) | ⨁⨁⨁◯  Moderate |
| HDL | 13 | randomised trials | not serious | not serious | not serious | serious^a^ | none | 410 | 336 | MD **0.37 higher**  (0.65 lower to 1.39 higher) | ⨁⨁⨁◯  Moderate |
| LDL | 13 | randomised trials | not serious | not serious | not serious | serious^a^ | none | 410 | 336 | MD **3.41 lower**  (9.01 lower to 2.18 higher) | ⨁⨁⨁◯  Moderate |
| SBP | 4 | randomised trials | not serious | not serious | not serious | not serious | none | 110 | 108 | MD **7.49 lower**  (11.67 lower to 3.3 lower) | ⨁⨁⨁⨁  High |
| DBP | 4 | randomised trials | not serious | not serious | not serious | not serious | none | 110 | 108 | MD **0.01 higher**  (3.4 lower to 3.42 higher) | ⨁⨁⨁⨁  High |
| BMI | 7 | randomised trials | not serious | not serious | not serious | not serious | none | 201 | 177 | MD **0.13 lower**  (0.53 lower to 0.26 higher) | ⨁⨁⨁⨁  High |

**CI:** confidence interval; **MD:** mean difference
