# Supplementary material for: Effects of Crocus sativus on glycemic control and cardiometabolic parameters among patients with metabolic syndrome and related disorders: a systematic review and meta-analysis of randomized controlled trials
Source: Nutr Metab (Lond). 2024 May 25;21:28. doi: 10.1186/s12986-024-00806-y (PMC11127410; doi:10.1186/s12986-024-00806-y)

	Crocus sativus			Placebo				Mean Difference
**Study or Subgroup**	**Mean**	**SD**	**Total**	**Mean**	**SD**	**Total**	**Weight**	**IV, Random, 95% CI**
Abedimanesh et al.(2017)(a)	-0.79	2.28	25	0.03	2.89	12	4.5%	-0.82 [-2.68, 1.04]
Abedimanesh et al.(2017)(b)	-0.29	2.55	25	0.03	2.89	13	4.5%	-0.32 [-2.18, 1.54]
Ebrahimi et al.(2019)	-0.49	1.01	40	-0.64	2.94	40	16.7%	0.15 [-0.81, 1.11]
Jaafarinia et al.(2022)	-0.46	1.25	21	-0.1	0.56	19	44.4%	-0.36 [-0.95, 0.23]
Karimi-Nazari et al.(2019)	-0.11	1.5	36	-0.14	2.01	39	24.3%	0.03 [-0.77, 0.83]
Kermani et al.(2017a)	0.5	3.8	24	-0.2	5.03	24	2.4%	0.70 [-1.82, 3.22]
Tajaddini et al.(2023)	0.7	4.25	30	-0.2	4.5	30	3.2%	0.90 [-1.31, 3.11]
**Total (95% CI)**			**201**			**177**	**100.0%**	**-0.13 [-0.53, 0.26]**

Heterogeneity: Tau² = 0.00; Chi² = 2.87, df = 6 (P = 0.82); I² = 0%

Test for overall effect: Z = 0.66 (P = 0.51)

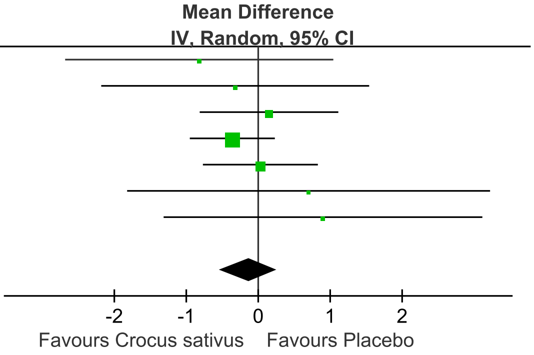

Supplement: Supplementary file 4 — Additional file 4: FigureS1a, FigureS1b, FigureS2, FigureS3a, FigureS3b, FigureS3c, FigureS4a, FigureS4b, FigureS4c, FigureS5- FigureS10. Forest plot of subgroup analysis by duration of intervention of the estimated impact of Crocus sativus on FBG. Forest plot of subgroup analysis by a dose of crocin of the estimated impact of Crocus sativus on FBG. Forest plot of subgroup analysis by duration of intervention of the estimated impact of Crocus sativus on HbA1c. Forest plot of the efficacy of Crocus sativus on FINS. Forest plot of subgroup analysis by saffron preparations of the estimated impact of Crocus sativus on FINS. Forest plot of subgroup analysis by duration of intervention of the estimated impact of Crocus sativus on FINS. Forest plot of the efficacy of Crocus sativus on HOMA-IR. Forest plot of subgroup analysis by saffron preparations of the estimated impact of Crocus sativus on HOMA-IR. Forest plot of subgroup analysis by duration of intervention of the estimated impact of Crocus sativus on HOMA-IR. Forest plot of the efficacy of Crocus sativus on TG. Forest plot of the efficacy of Crocus sativus on TC. Forest plot of subgroup analysis by type of chronic condition of the estimated impact of Crocus sativus on HDL. Forest plot of the efficacy of Crocus sativus on LDL. Forest plot of the efficacy of Crocus sativus on DBP. Forest plot of the efficacy of Crocus sativus on BMI. [file 12986_2024_806_MOESM4_ESM.zip › Additional file 4/FigureS10.pdf]
