# Supplementary figures and images for: Effects of Crocus sativus on glycemic control and cardiometabolic parameters among patients with metabolic syndrome and related disorders: a systematic review and meta-analysis of randomized controlled trials
Source: Nutr Metab (Lond). 2024 May 25;21:28. doi: 10.1186/s12986-024-00806-y (PMC11127410; doi:10.1186/s12986-024-00806-y)

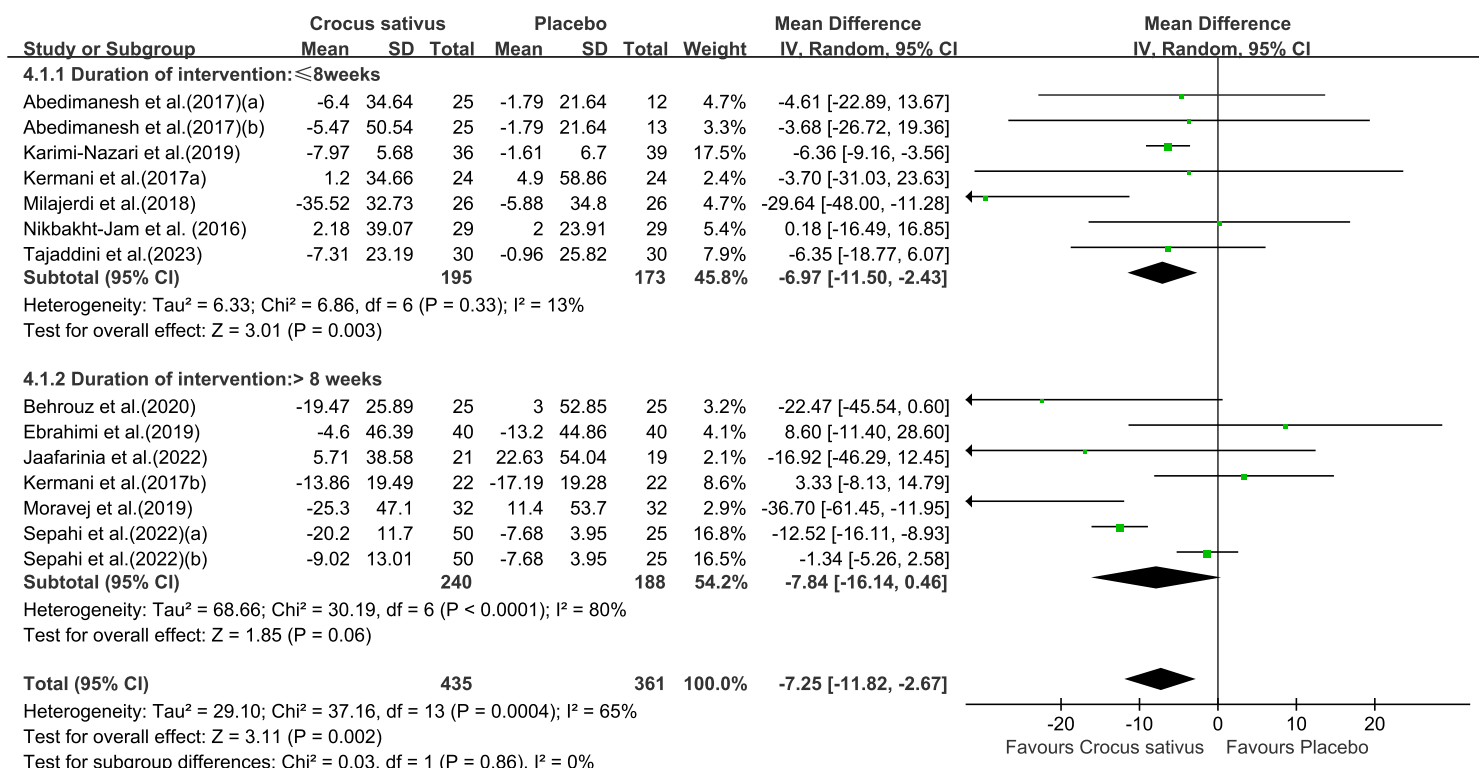

Supplement: Supplementary file 4 — Additional file 4: FigureS1a, FigureS1b, FigureS2, FigureS3a, FigureS3b, FigureS3c, FigureS4a, FigureS4b, FigureS4c, FigureS5- FigureS10. Forest plot of subgroup analysis by duration of intervention of the estimated impact of Crocus sativus on FBG. Forest plot of subgroup analysis by a dose of crocin of the estimated impact of Crocus sativus on FBG. Forest plot of subgroup analysis by duration of intervention of the estimated impact of Crocus sativus on HbA1c. Forest plot of the efficacy of Crocus sativus on FINS. Forest plot of subgroup analysis by saffron preparations of the estimated impact of Crocus sativus on FINS. Forest plot of subgroup analysis by duration of intervention of the estimated impact of Crocus sativus on FINS. Forest plot of the efficacy of Crocus sativus on HOMA-IR. Forest plot of subgroup analysis by saffron preparations of the estimated impact of Crocus sativus on HOMA-IR. Forest plot of subgroup analysis by duration of intervention of the estimated impact of Crocus sativus on HOMA-IR. Forest plot of the efficacy of Crocus sativus on TG. Forest plot of the efficacy of Crocus sativus on TC. Forest plot of subgroup analysis by type of chronic condition of the estimated impact of Crocus sativus on HDL. Forest plot of the efficacy of Crocus sativus on LDL. Forest plot of the efficacy of Crocus sativus on DBP. Forest plot of the efficacy of Crocus sativus on BMI. [file 12986_2024_806_MOESM4_ESM.zip › Additional file 4/FigureS1a.pdf]

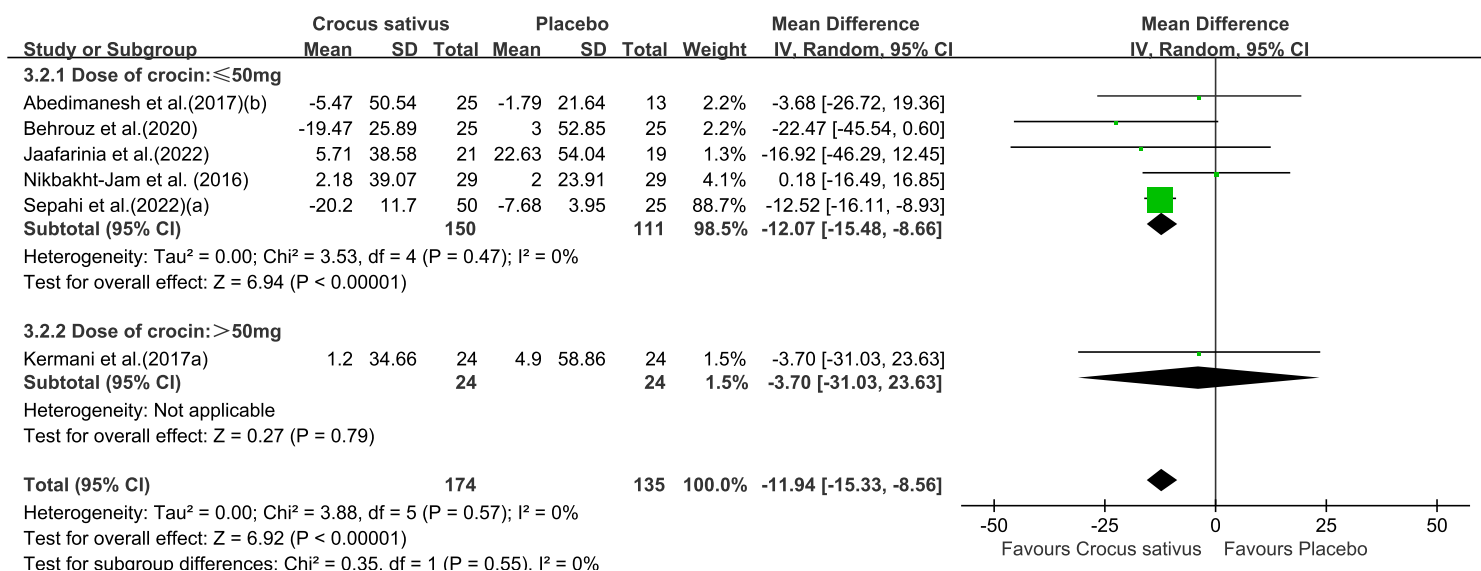

Supplement: Supplementary file 4 — Additional file 4: FigureS1a, FigureS1b, FigureS2, FigureS3a, FigureS3b, FigureS3c, FigureS4a, FigureS4b, FigureS4c, FigureS5- FigureS10. Forest plot of subgroup analysis by duration of intervention of the estimated impact of Crocus sativus on FBG. Forest plot of subgroup analysis by a dose of crocin of the estimated impact of Crocus sativus on FBG. Forest plot of subgroup analysis by duration of intervention of the estimated impact of Crocus sativus on HbA1c. Forest plot of the efficacy of Crocus sativus on FINS. Forest plot of subgroup analysis by saffron preparations of the estimated impact of Crocus sativus on FINS. Forest plot of subgroup analysis by duration of intervention of the estimated impact of Crocus sativus on FINS. Forest plot of the efficacy of Crocus sativus on HOMA-IR. Forest plot of subgroup analysis by saffron preparations of the estimated impact of Crocus sativus on HOMA-IR. Forest plot of subgroup analysis by duration of intervention of the estimated impact of Crocus sativus on HOMA-IR. Forest plot of the efficacy of Crocus sativus on TG. Forest plot of the efficacy of Crocus sativus on TC. Forest plot of subgroup analysis by type of chronic condition of the estimated impact of Crocus sativus on HDL. Forest plot of the efficacy of Crocus sativus on LDL. Forest plot of the efficacy of Crocus sativus on DBP. Forest plot of the efficacy of Crocus sativus on BMI. [file 12986_2024_806_MOESM4_ESM.zip › Additional file 4/FigureS1b.pdf]

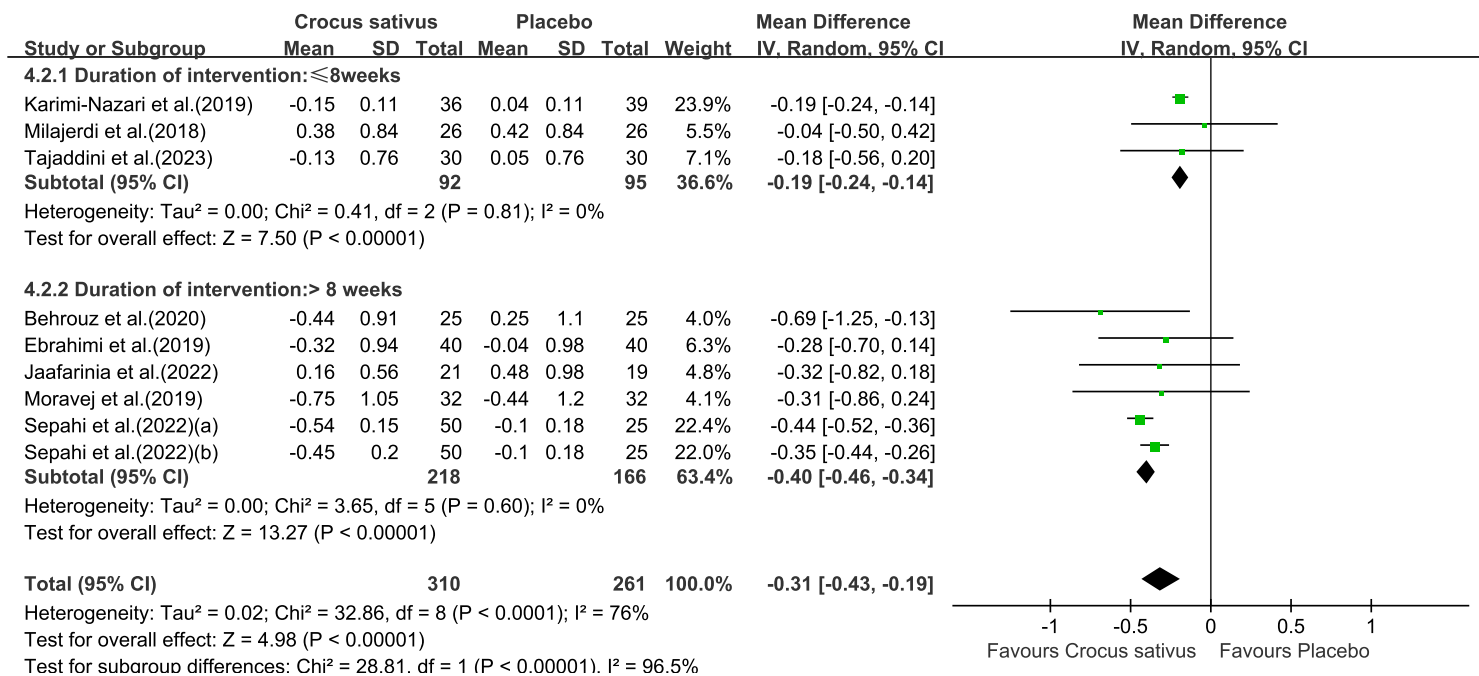

Supplement: Supplementary file 4 — Additional file 4: FigureS1a, FigureS1b, FigureS2, FigureS3a, FigureS3b, FigureS3c, FigureS4a, FigureS4b, FigureS4c, FigureS5- FigureS10. Forest plot of subgroup analysis by duration of intervention of the estimated impact of Crocus sativus on FBG. Forest plot of subgroup analysis by a dose of crocin of the estimated impact of Crocus sativus on FBG. Forest plot of subgroup analysis by duration of intervention of the estimated impact of Crocus sativus on HbA1c. Forest plot of the efficacy of Crocus sativus on FINS. Forest plot of subgroup analysis by saffron preparations of the estimated impact of Crocus sativus on FINS. Forest plot of subgroup analysis by duration of intervention of the estimated impact of Crocus sativus on FINS. Forest plot of the efficacy of Crocus sativus on HOMA-IR. Forest plot of subgroup analysis by saffron preparations of the estimated impact of Crocus sativus on HOMA-IR. Forest plot of subgroup analysis by duration of intervention of the estimated impact of Crocus sativus on HOMA-IR. Forest plot of the efficacy of Crocus sativus on TG. Forest plot of the efficacy of Crocus sativus on TC. Forest plot of subgroup analysis by type of chronic condition of the estimated impact of Crocus sativus on HDL. Forest plot of the efficacy of Crocus sativus on LDL. Forest plot of the efficacy of Crocus sativus on DBP. Forest plot of the efficacy of Crocus sativus on BMI. [file 12986_2024_806_MOESM4_ESM.zip › Additional file 4/FigureS2.pdf]

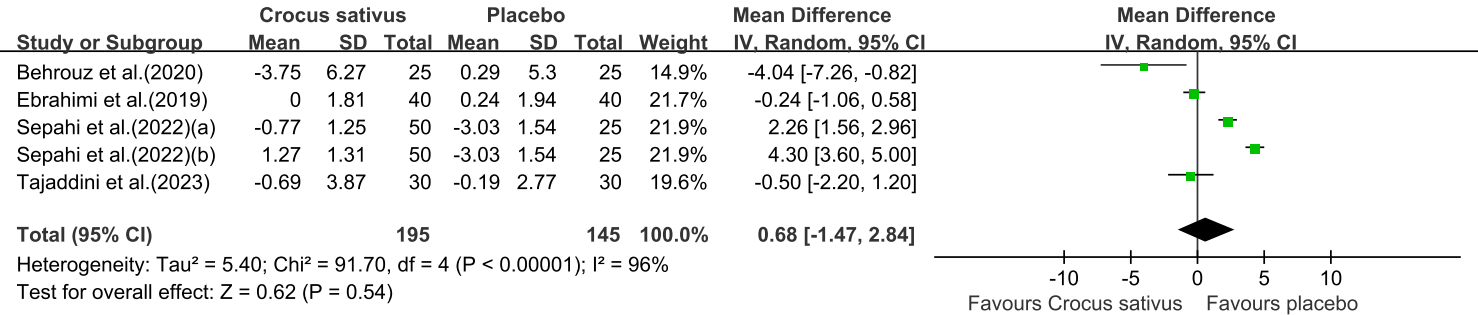

Supplement: Supplementary file 4 — Additional file 4: FigureS1a, FigureS1b, FigureS2, FigureS3a, FigureS3b, FigureS3c, FigureS4a, FigureS4b, FigureS4c, FigureS5- FigureS10. Forest plot of subgroup analysis by duration of intervention of the estimated impact of Crocus sativus on FBG. Forest plot of subgroup analysis by a dose of crocin of the estimated impact of Crocus sativus on FBG. Forest plot of subgroup analysis by duration of intervention of the estimated impact of Crocus sativus on HbA1c. Forest plot of the efficacy of Crocus sativus on FINS. Forest plot of subgroup analysis by saffron preparations of the estimated impact of Crocus sativus on FINS. Forest plot of subgroup analysis by duration of intervention of the estimated impact of Crocus sativus on FINS. Forest plot of the efficacy of Crocus sativus on HOMA-IR. Forest plot of subgroup analysis by saffron preparations of the estimated impact of Crocus sativus on HOMA-IR. Forest plot of subgroup analysis by duration of intervention of the estimated impact of Crocus sativus on HOMA-IR. Forest plot of the efficacy of Crocus sativus on TG. Forest plot of the efficacy of Crocus sativus on TC. Forest plot of subgroup analysis by type of chronic condition of the estimated impact of Crocus sativus on HDL. Forest plot of the efficacy of Crocus sativus on LDL. Forest plot of the efficacy of Crocus sativus on DBP. Forest plot of the efficacy of Crocus sativus on BMI. [file 12986_2024_806_MOESM4_ESM.zip › Additional file 4/FigureS3a.pdf]

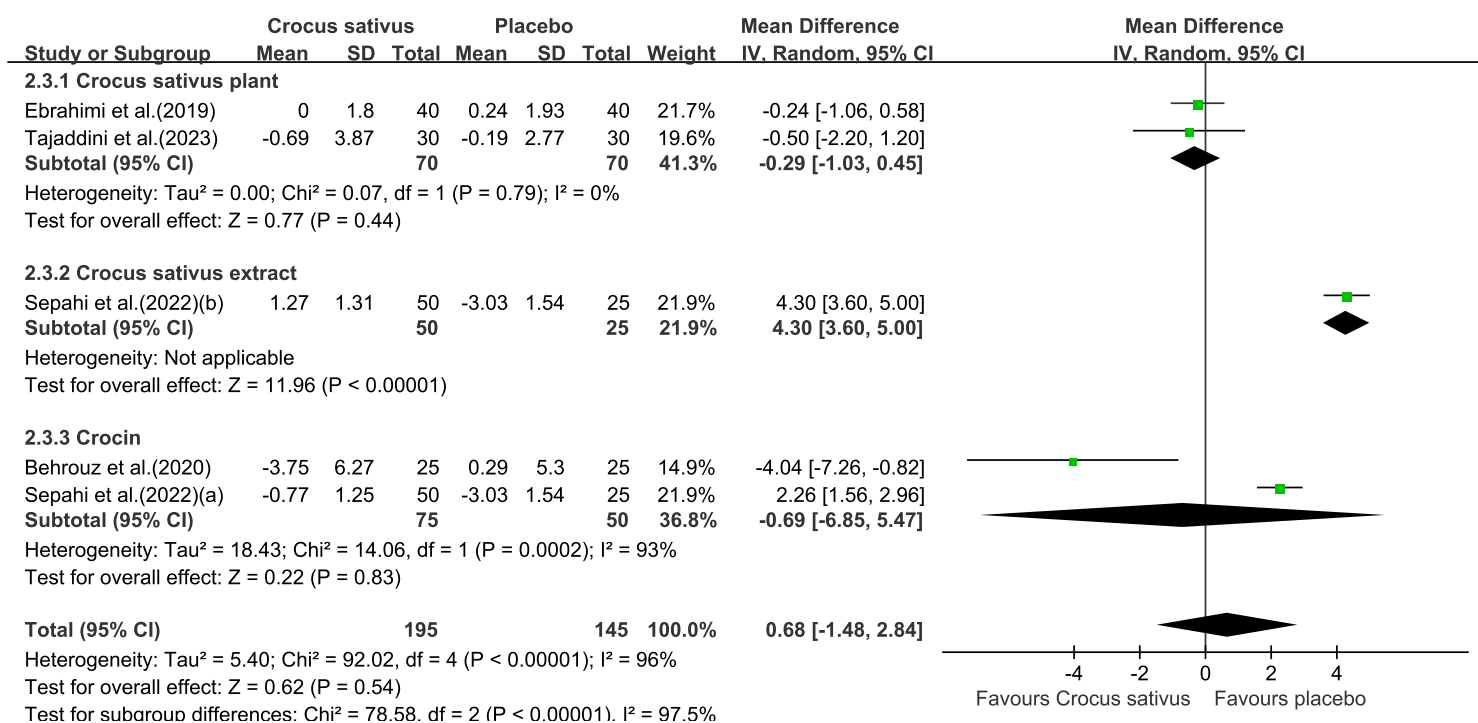

Supplement: Supplementary file 4 — Additional file 4: FigureS1a, FigureS1b, FigureS2, FigureS3a, FigureS3b, FigureS3c, FigureS4a, FigureS4b, FigureS4c, FigureS5- FigureS10. Forest plot of subgroup analysis by duration of intervention of the estimated impact of Crocus sativus on FBG. Forest plot of subgroup analysis by a dose of crocin of the estimated impact of Crocus sativus on FBG. Forest plot of subgroup analysis by duration of intervention of the estimated impact of Crocus sativus on HbA1c. Forest plot of the efficacy of Crocus sativus on FINS. Forest plot of subgroup analysis by saffron preparations of the estimated impact of Crocus sativus on FINS. Forest plot of subgroup analysis by duration of intervention of the estimated impact of Crocus sativus on FINS. Forest plot of the efficacy of Crocus sativus on HOMA-IR. Forest plot of subgroup analysis by saffron preparations of the estimated impact of Crocus sativus on HOMA-IR. Forest plot of subgroup analysis by duration of intervention of the estimated impact of Crocus sativus on HOMA-IR. Forest plot of the efficacy of Crocus sativus on TG. Forest plot of the efficacy of Crocus sativus on TC. Forest plot of subgroup analysis by type of chronic condition of the estimated impact of Crocus sativus on HDL. Forest plot of the efficacy of Crocus sativus on LDL. Forest plot of the efficacy of Crocus sativus on DBP. Forest plot of the efficacy of Crocus sativus on BMI. [file 12986_2024_806_MOESM4_ESM.zip › Additional file 4/FigureS3b.pdf]

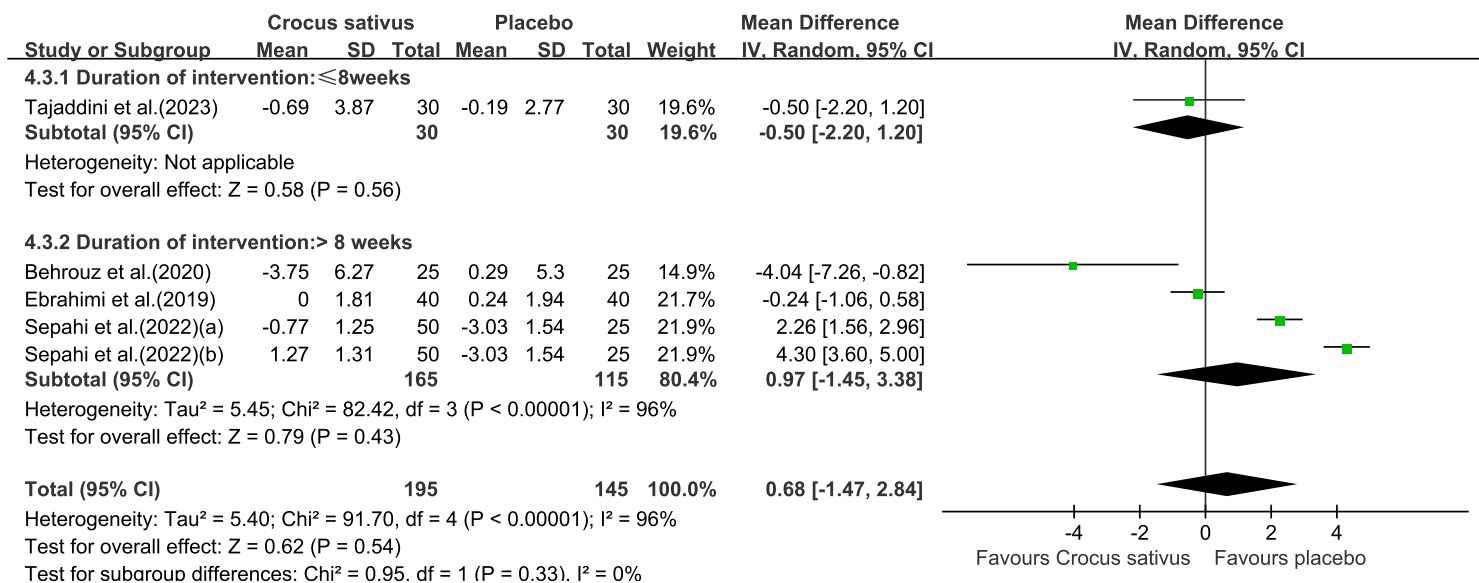

Supplement: Supplementary file 4 — Additional file 4: FigureS1a, FigureS1b, FigureS2, FigureS3a, FigureS3b, FigureS3c, FigureS4a, FigureS4b, FigureS4c, FigureS5- FigureS10. Forest plot of subgroup analysis by duration of intervention of the estimated impact of Crocus sativus on FBG. Forest plot of subgroup analysis by a dose of crocin of the estimated impact of Crocus sativus on FBG. Forest plot of subgroup analysis by duration of intervention of the estimated impact of Crocus sativus on HbA1c. Forest plot of the efficacy of Crocus sativus on FINS. Forest plot of subgroup analysis by saffron preparations of the estimated impact of Crocus sativus on FINS. Forest plot of subgroup analysis by duration of intervention of the estimated impact of Crocus sativus on FINS. Forest plot of the efficacy of Crocus sativus on HOMA-IR. Forest plot of subgroup analysis by saffron preparations of the estimated impact of Crocus sativus on HOMA-IR. Forest plot of subgroup analysis by duration of intervention of the estimated impact of Crocus sativus on HOMA-IR. Forest plot of the efficacy of Crocus sativus on TG. Forest plot of the efficacy of Crocus sativus on TC. Forest plot of subgroup analysis by type of chronic condition of the estimated impact of Crocus sativus on HDL. Forest plot of the efficacy of Crocus sativus on LDL. Forest plot of the efficacy of Crocus sativus on DBP. Forest plot of the efficacy of Crocus sativus on BMI. [file 12986_2024_806_MOESM4_ESM.zip › Additional file 4/FigureS3c.pdf]

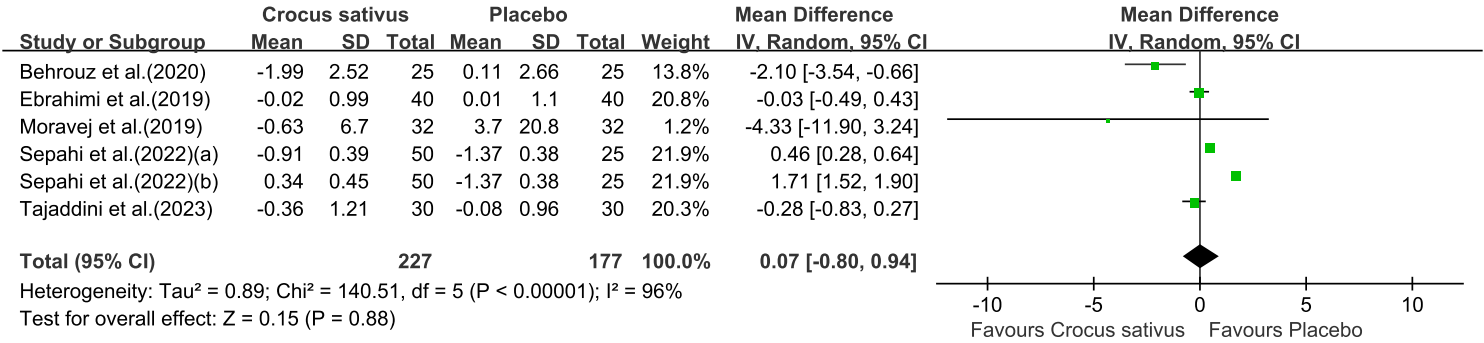

Supplement: Supplementary file 4 — Additional file 4: FigureS1a, FigureS1b, FigureS2, FigureS3a, FigureS3b, FigureS3c, FigureS4a, FigureS4b, FigureS4c, FigureS5- FigureS10. Forest plot of subgroup analysis by duration of intervention of the estimated impact of Crocus sativus on FBG. Forest plot of subgroup analysis by a dose of crocin of the estimated impact of Crocus sativus on FBG. Forest plot of subgroup analysis by duration of intervention of the estimated impact of Crocus sativus on HbA1c. Forest plot of the efficacy of Crocus sativus on FINS. Forest plot of subgroup analysis by saffron preparations of the estimated impact of Crocus sativus on FINS. Forest plot of subgroup analysis by duration of intervention of the estimated impact of Crocus sativus on FINS. Forest plot of the efficacy of Crocus sativus on HOMA-IR. Forest plot of subgroup analysis by saffron preparations of the estimated impact of Crocus sativus on HOMA-IR. Forest plot of subgroup analysis by duration of intervention of the estimated impact of Crocus sativus on HOMA-IR. Forest plot of the efficacy of Crocus sativus on TG. Forest plot of the efficacy of Crocus sativus on TC. Forest plot of subgroup analysis by type of chronic condition of the estimated impact of Crocus sativus on HDL. Forest plot of the efficacy of Crocus sativus on LDL. Forest plot of the efficacy of Crocus sativus on DBP. Forest plot of the efficacy of Crocus sativus on BMI. [file 12986_2024_806_MOESM4_ESM.zip › Additional file 4/FigureS4a.pdf]

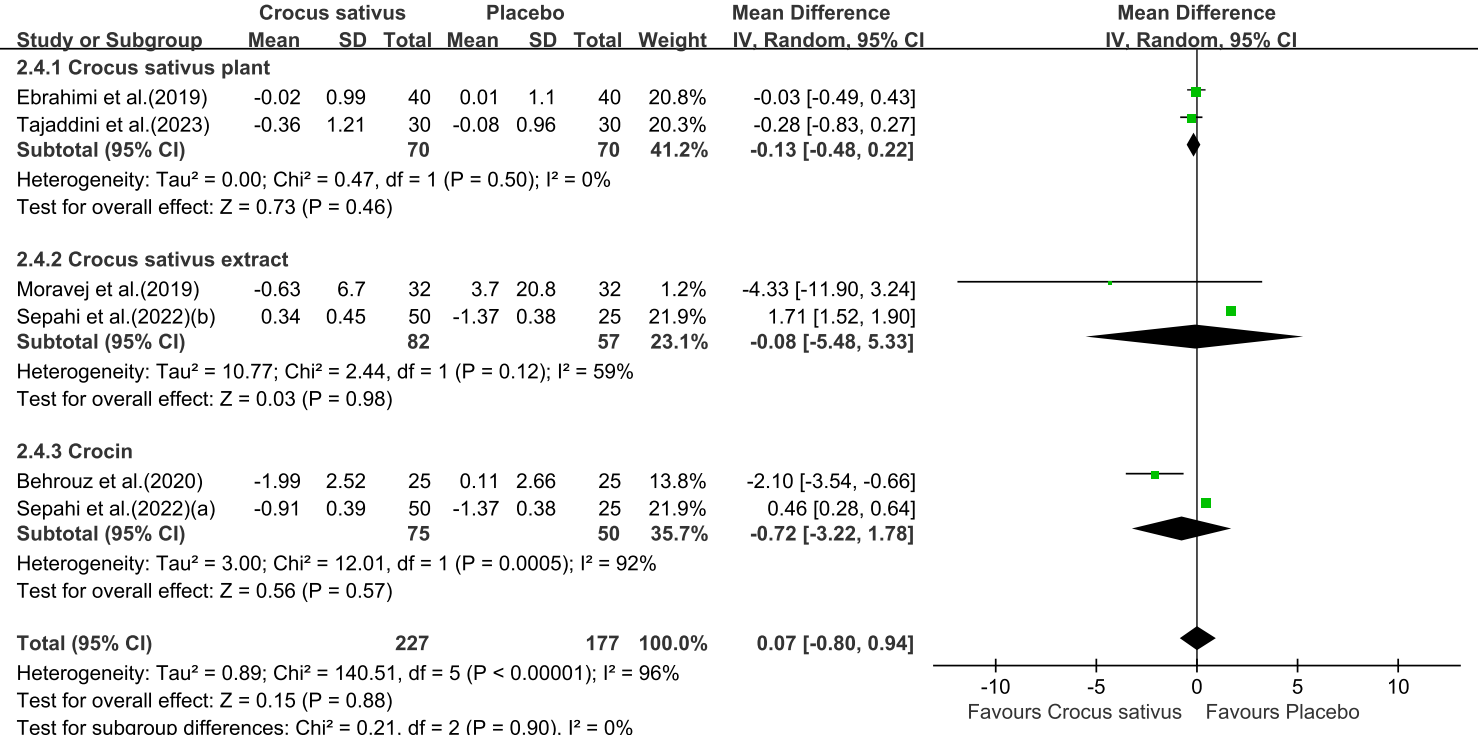

Supplement: Supplementary file 4 — Additional file 4: FigureS1a, FigureS1b, FigureS2, FigureS3a, FigureS3b, FigureS3c, FigureS4a, FigureS4b, FigureS4c, FigureS5- FigureS10. Forest plot of subgroup analysis by duration of intervention of the estimated impact of Crocus sativus on FBG. Forest plot of subgroup analysis by a dose of crocin of the estimated impact of Crocus sativus on FBG. Forest plot of subgroup analysis by duration of intervention of the estimated impact of Crocus sativus on HbA1c. Forest plot of the efficacy of Crocus sativus on FINS. Forest plot of subgroup analysis by saffron preparations of the estimated impact of Crocus sativus on FINS. Forest plot of subgroup analysis by duration of intervention of the estimated impact of Crocus sativus on FINS. Forest plot of the efficacy of Crocus sativus on HOMA-IR. Forest plot of subgroup analysis by saffron preparations of the estimated impact of Crocus sativus on HOMA-IR. Forest plot of subgroup analysis by duration of intervention of the estimated impact of Crocus sativus on HOMA-IR. Forest plot of the efficacy of Crocus sativus on TG. Forest plot of the efficacy of Crocus sativus on TC. Forest plot of subgroup analysis by type of chronic condition of the estimated impact of Crocus sativus on HDL. Forest plot of the efficacy of Crocus sativus on LDL. Forest plot of the efficacy of Crocus sativus on DBP. Forest plot of the efficacy of Crocus sativus on BMI. [file 12986_2024_806_MOESM4_ESM.zip › Additional file 4/FigureS4b.pdf]

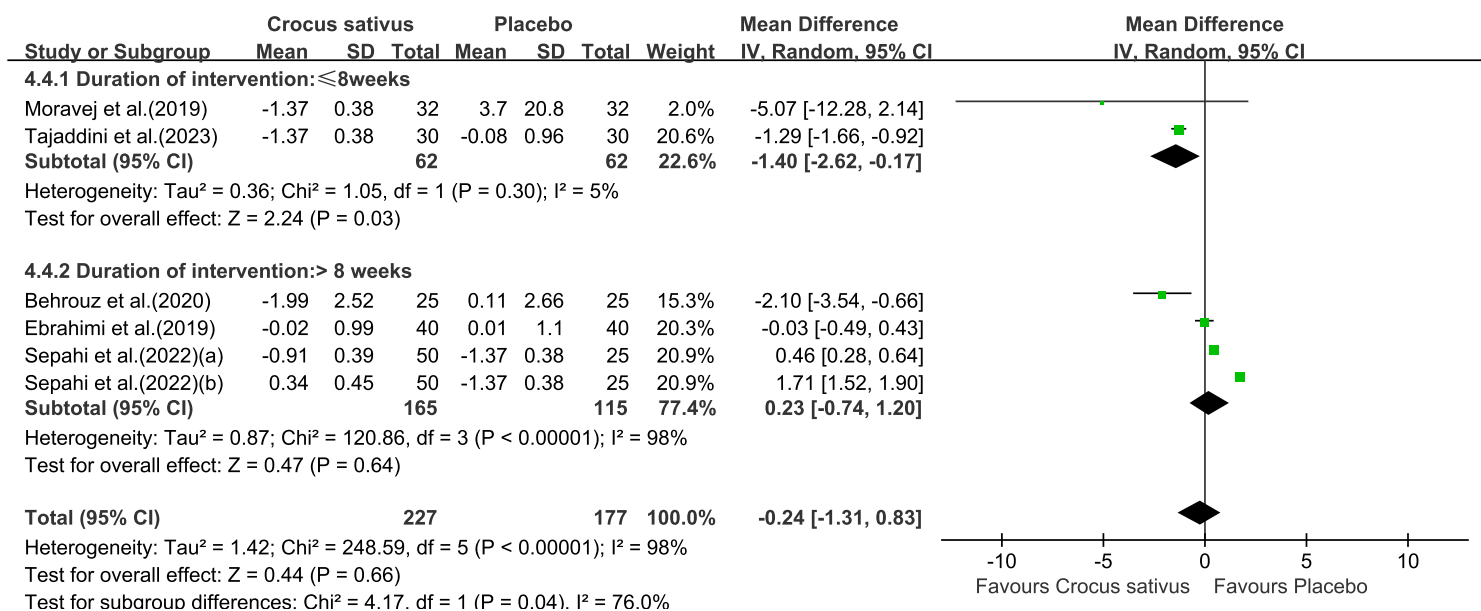

Supplement: Supplementary file 4 — Additional file 4: FigureS1a, FigureS1b, FigureS2, FigureS3a, FigureS3b, FigureS3c, FigureS4a, FigureS4b, FigureS4c, FigureS5- FigureS10. Forest plot of subgroup analysis by duration of intervention of the estimated impact of Crocus sativus on FBG. Forest plot of subgroup analysis by a dose of crocin of the estimated impact of Crocus sativus on FBG. Forest plot of subgroup analysis by duration of intervention of the estimated impact of Crocus sativus on HbA1c. Forest plot of the efficacy of Crocus sativus on FINS. Forest plot of subgroup analysis by saffron preparations of the estimated impact of Crocus sativus on FINS. Forest plot of subgroup analysis by duration of intervention of the estimated impact of Crocus sativus on FINS. Forest plot of the efficacy of Crocus sativus on HOMA-IR. Forest plot of subgroup analysis by saffron preparations of the estimated impact of Crocus sativus on HOMA-IR. Forest plot of subgroup analysis by duration of intervention of the estimated impact of Crocus sativus on HOMA-IR. Forest plot of the efficacy of Crocus sativus on TG. Forest plot of the efficacy of Crocus sativus on TC. Forest plot of subgroup analysis by type of chronic condition of the estimated impact of Crocus sativus on HDL. Forest plot of the efficacy of Crocus sativus on LDL. Forest plot of the efficacy of Crocus sativus on DBP. Forest plot of the efficacy of Crocus sativus on BMI. [file 12986_2024_806_MOESM4_ESM.zip › Additional file 4/FigureS4c.pdf]

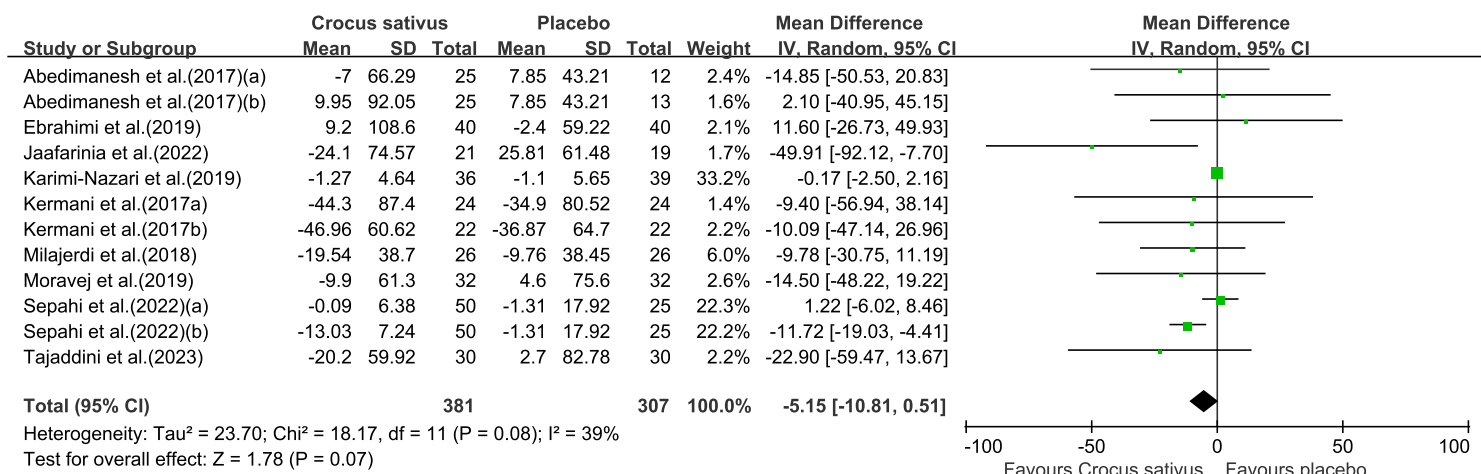

Supplement: Supplementary file 4 — Additional file 4: FigureS1a, FigureS1b, FigureS2, FigureS3a, FigureS3b, FigureS3c, FigureS4a, FigureS4b, FigureS4c, FigureS5- FigureS10. Forest plot of subgroup analysis by duration of intervention of the estimated impact of Crocus sativus on FBG. Forest plot of subgroup analysis by a dose of crocin of the estimated impact of Crocus sativus on FBG. Forest plot of subgroup analysis by duration of intervention of the estimated impact of Crocus sativus on HbA1c. Forest plot of the efficacy of Crocus sativus on FINS. Forest plot of subgroup analysis by saffron preparations of the estimated impact of Crocus sativus on FINS. Forest plot of subgroup analysis by duration of intervention of the estimated impact of Crocus sativus on FINS. Forest plot of the efficacy of Crocus sativus on HOMA-IR. Forest plot of subgroup analysis by saffron preparations of the estimated impact of Crocus sativus on HOMA-IR. Forest plot of subgroup analysis by duration of intervention of the estimated impact of Crocus sativus on HOMA-IR. Forest plot of the efficacy of Crocus sativus on TG. Forest plot of the efficacy of Crocus sativus on TC. Forest plot of subgroup analysis by type of chronic condition of the estimated impact of Crocus sativus on HDL. Forest plot of the efficacy of Crocus sativus on LDL. Forest plot of the efficacy of Crocus sativus on DBP. Forest plot of the efficacy of Crocus sativus on BMI. [file 12986_2024_806_MOESM4_ESM.zip › Additional file 4/FigureS5.pdf]

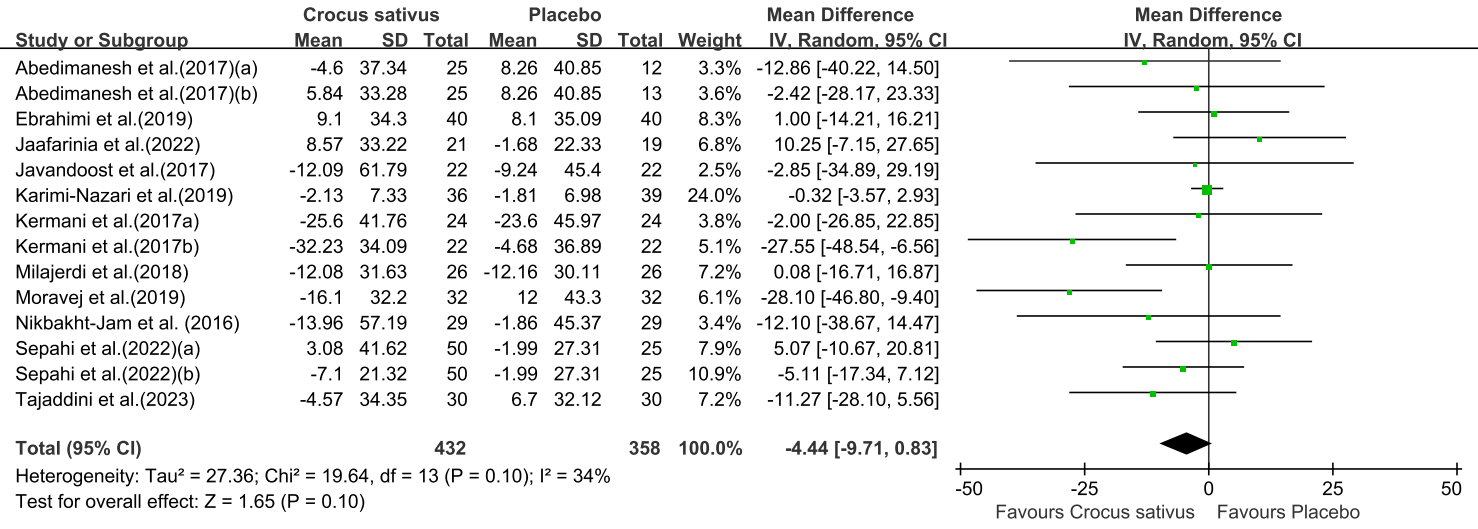

Supplement: Supplementary file 4 — Additional file 4: FigureS1a, FigureS1b, FigureS2, FigureS3a, FigureS3b, FigureS3c, FigureS4a, FigureS4b, FigureS4c, FigureS5- FigureS10. Forest plot of subgroup analysis by duration of intervention of the estimated impact of Crocus sativus on FBG. Forest plot of subgroup analysis by a dose of crocin of the estimated impact of Crocus sativus on FBG. Forest plot of subgroup analysis by duration of intervention of the estimated impact of Crocus sativus on HbA1c. Forest plot of the efficacy of Crocus sativus on FINS. Forest plot of subgroup analysis by saffron preparations of the estimated impact of Crocus sativus on FINS. Forest plot of subgroup analysis by duration of intervention of the estimated impact of Crocus sativus on FINS. Forest plot of the efficacy of Crocus sativus on HOMA-IR. Forest plot of subgroup analysis by saffron preparations of the estimated impact of Crocus sativus on HOMA-IR. Forest plot of subgroup analysis by duration of intervention of the estimated impact of Crocus sativus on HOMA-IR. Forest plot of the efficacy of Crocus sativus on TG. Forest plot of the efficacy of Crocus sativus on TC. Forest plot of subgroup analysis by type of chronic condition of the estimated impact of Crocus sativus on HDL. Forest plot of the efficacy of Crocus sativus on LDL. Forest plot of the efficacy of Crocus sativus on DBP. Forest plot of the efficacy of Crocus sativus on BMI. [file 12986_2024_806_MOESM4_ESM.zip › Additional file 4/FigureS6.pdf]

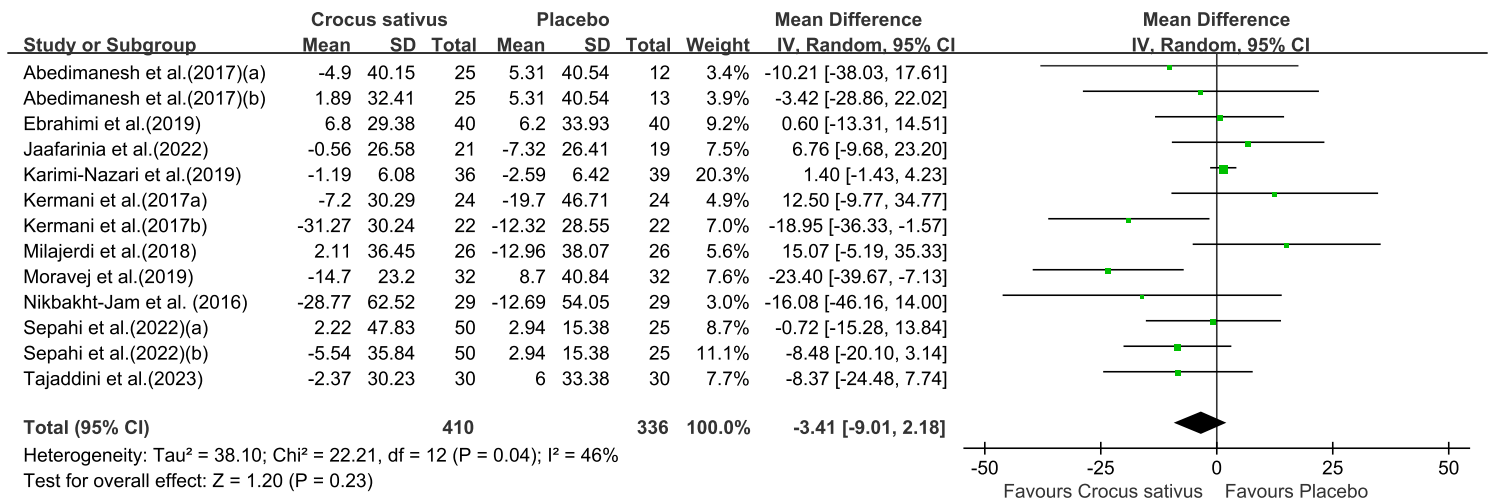

Supplement: Supplementary file 4 — Additional file 4: FigureS1a, FigureS1b, FigureS2, FigureS3a, FigureS3b, FigureS3c, FigureS4a, FigureS4b, FigureS4c, FigureS5- FigureS10. Forest plot of subgroup analysis by duration of intervention of the estimated impact of Crocus sativus on FBG. Forest plot of subgroup analysis by a dose of crocin of the estimated impact of Crocus sativus on FBG. Forest plot of subgroup analysis by duration of intervention of the estimated impact of Crocus sativus on HbA1c. Forest plot of the efficacy of Crocus sativus on FINS. Forest plot of subgroup analysis by saffron preparations of the estimated impact of Crocus sativus on FINS. Forest plot of subgroup analysis by duration of intervention of the estimated impact of Crocus sativus on FINS. Forest plot of the efficacy of Crocus sativus on HOMA-IR. Forest plot of subgroup analysis by saffron preparations of the estimated impact of Crocus sativus on HOMA-IR. Forest plot of subgroup analysis by duration of intervention of the estimated impact of Crocus sativus on HOMA-IR. Forest plot of the efficacy of Crocus sativus on TG. Forest plot of the efficacy of Crocus sativus on TC. Forest plot of subgroup analysis by type of chronic condition of the estimated impact of Crocus sativus on HDL. Forest plot of the efficacy of Crocus sativus on LDL. Forest plot of the efficacy of Crocus sativus on DBP. Forest plot of the efficacy of Crocus sativus on BMI. [file 12986_2024_806_MOESM4_ESM.zip › Additional file 4/FigureS7.pdf]

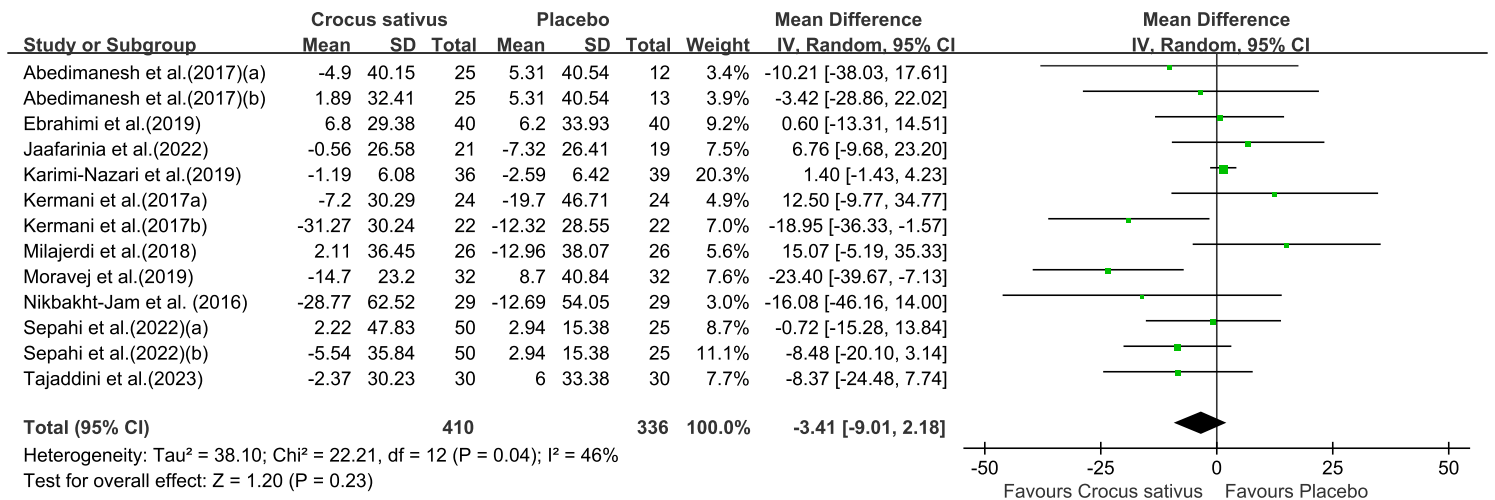

Supplement: Supplementary file 4 — Additional file 4: FigureS1a, FigureS1b, FigureS2, FigureS3a, FigureS3b, FigureS3c, FigureS4a, FigureS4b, FigureS4c, FigureS5- FigureS10. Forest plot of subgroup analysis by duration of intervention of the estimated impact of Crocus sativus on FBG. Forest plot of subgroup analysis by a dose of crocin of the estimated impact of Crocus sativus on FBG. Forest plot of subgroup analysis by duration of intervention of the estimated impact of Crocus sativus on HbA1c. Forest plot of the efficacy of Crocus sativus on FINS. Forest plot of subgroup analysis by saffron preparations of the estimated impact of Crocus sativus on FINS. Forest plot of subgroup analysis by duration of intervention of the estimated impact of Crocus sativus on FINS. Forest plot of the efficacy of Crocus sativus on HOMA-IR. Forest plot of subgroup analysis by saffron preparations of the estimated impact of Crocus sativus on HOMA-IR. Forest plot of subgroup analysis by duration of intervention of the estimated impact of Crocus sativus on HOMA-IR. Forest plot of the efficacy of Crocus sativus on TG. Forest plot of the efficacy of Crocus sativus on TC. Forest plot of subgroup analysis by type of chronic condition of the estimated impact of Crocus sativus on HDL. Forest plot of the efficacy of Crocus sativus on LDL. Forest plot of the efficacy of Crocus sativus on DBP. Forest plot of the efficacy of Crocus sativus on BMI. [file 12986_2024_806_MOESM4_ESM.zip › Additional file 4/FigureS8.pdf]

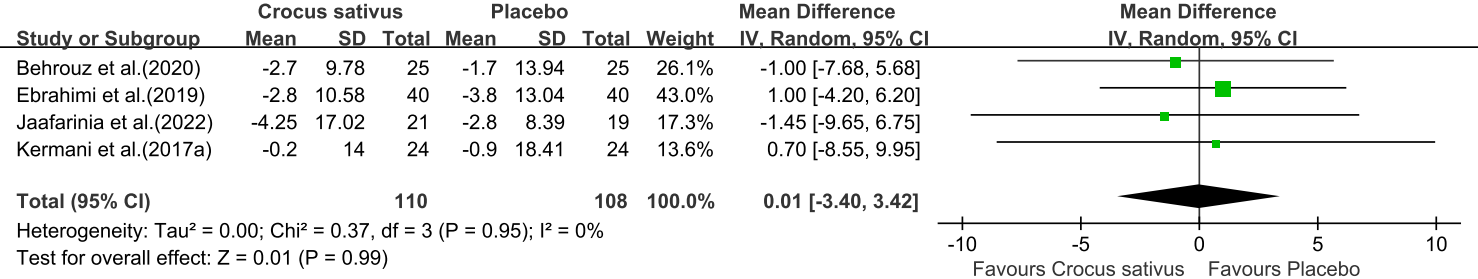

Supplement: Supplementary file 4 — Additional file 4: FigureS1a, FigureS1b, FigureS2, FigureS3a, FigureS3b, FigureS3c, FigureS4a, FigureS4b, FigureS4c, FigureS5- FigureS10. Forest plot of subgroup analysis by duration of intervention of the estimated impact of Crocus sativus on FBG. Forest plot of subgroup analysis by a dose of crocin of the estimated impact of Crocus sativus on FBG. Forest plot of subgroup analysis by duration of intervention of the estimated impact of Crocus sativus on HbA1c. Forest plot of the efficacy of Crocus sativus on FINS. Forest plot of subgroup analysis by saffron preparations of the estimated impact of Crocus sativus on FINS. Forest plot of subgroup analysis by duration of intervention of the estimated impact of Crocus sativus on FINS. Forest plot of the efficacy of Crocus sativus on HOMA-IR. Forest plot of subgroup analysis by saffron preparations of the estimated impact of Crocus sativus on HOMA-IR. Forest plot of subgroup analysis by duration of intervention of the estimated impact of Crocus sativus on HOMA-IR. Forest plot of the efficacy of Crocus sativus on TG. Forest plot of the efficacy of Crocus sativus on TC. Forest plot of subgroup analysis by type of chronic condition of the estimated impact of Crocus sativus on HDL. Forest plot of the efficacy of Crocus sativus on LDL. Forest plot of the efficacy of Crocus sativus on DBP. Forest plot of the efficacy of Crocus sativus on BMI. [file 12986_2024_806_MOESM4_ESM.zip › Additional file 4/FigureS9.pdf]

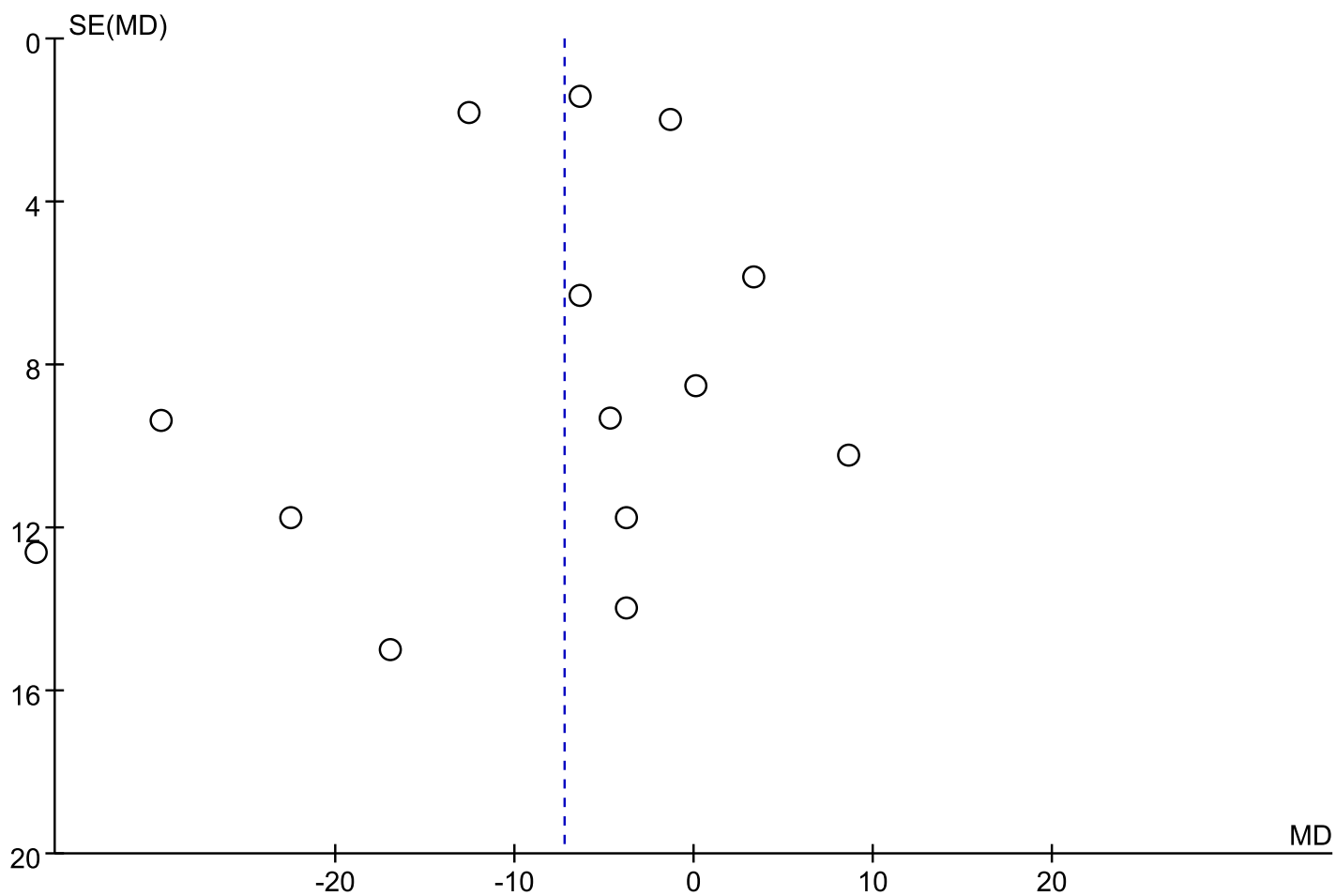

Supplement: Supplementary file 5 — Additional file 5: Figure S11a-Figure S11k. Funnel plot of FBG. Funnel plot of HbA1c. Funnel plot of FINS. Funnel plot of HOMA-IR. Funnel plot of TG. Funnel plot of TC. Funnel plot of HDL. Funnel plot of LDL. Funnel plot of SBP. Funnel plot of DBP. Funnel plot of BMI. [file 12986_2024_806_MOESM5_ESM.zip › Additional file 5/Figure S11a.pdf]

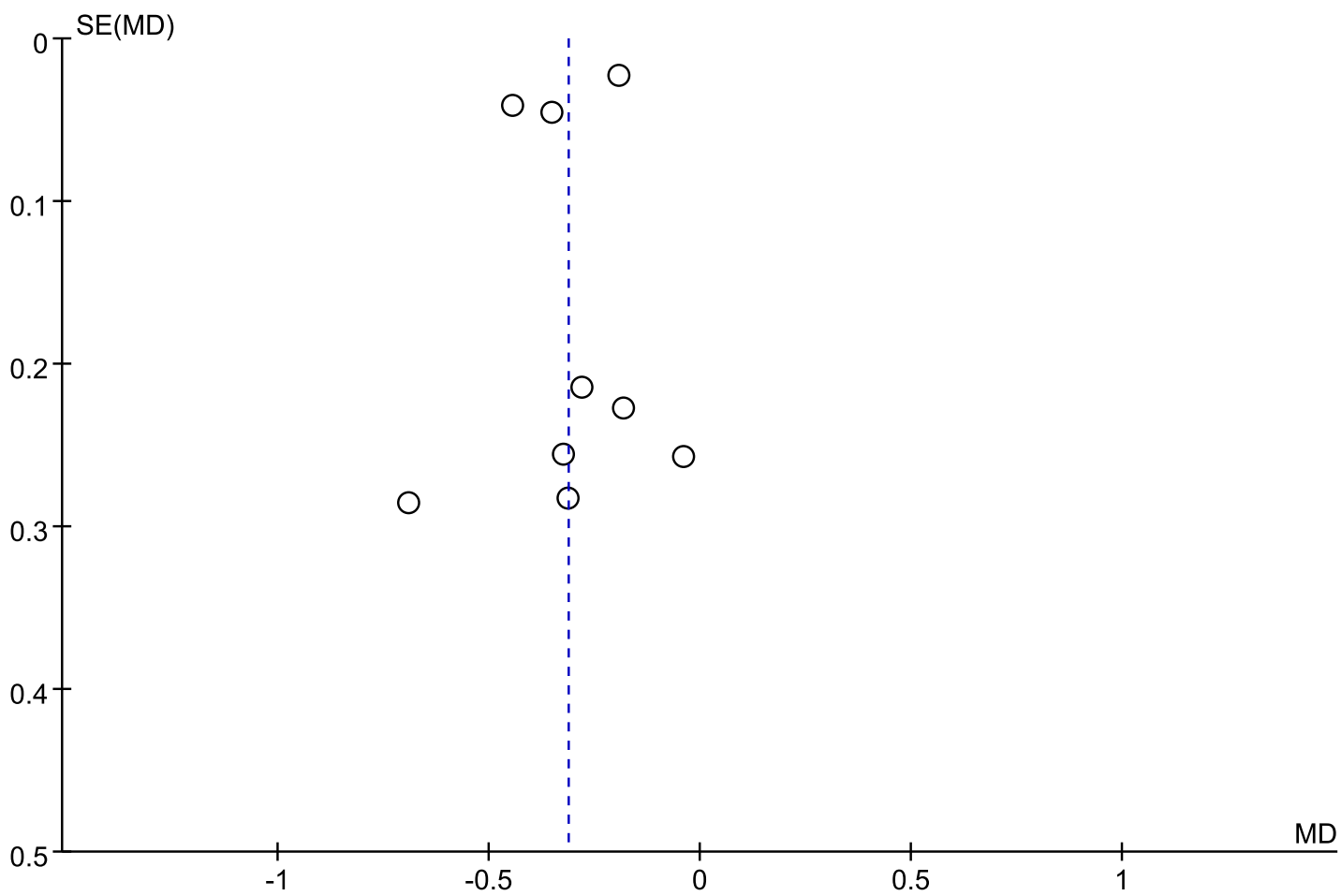

Supplement: Supplementary file 5 — Additional file 5: Figure S11a-Figure S11k. Funnel plot of FBG. Funnel plot of HbA1c. Funnel plot of FINS. Funnel plot of HOMA-IR. Funnel plot of TG. Funnel plot of TC. Funnel plot of HDL. Funnel plot of LDL. Funnel plot of SBP. Funnel plot of DBP. Funnel plot of BMI. [file 12986_2024_806_MOESM5_ESM.zip › Additional file 5/Figure S11b.pdf]

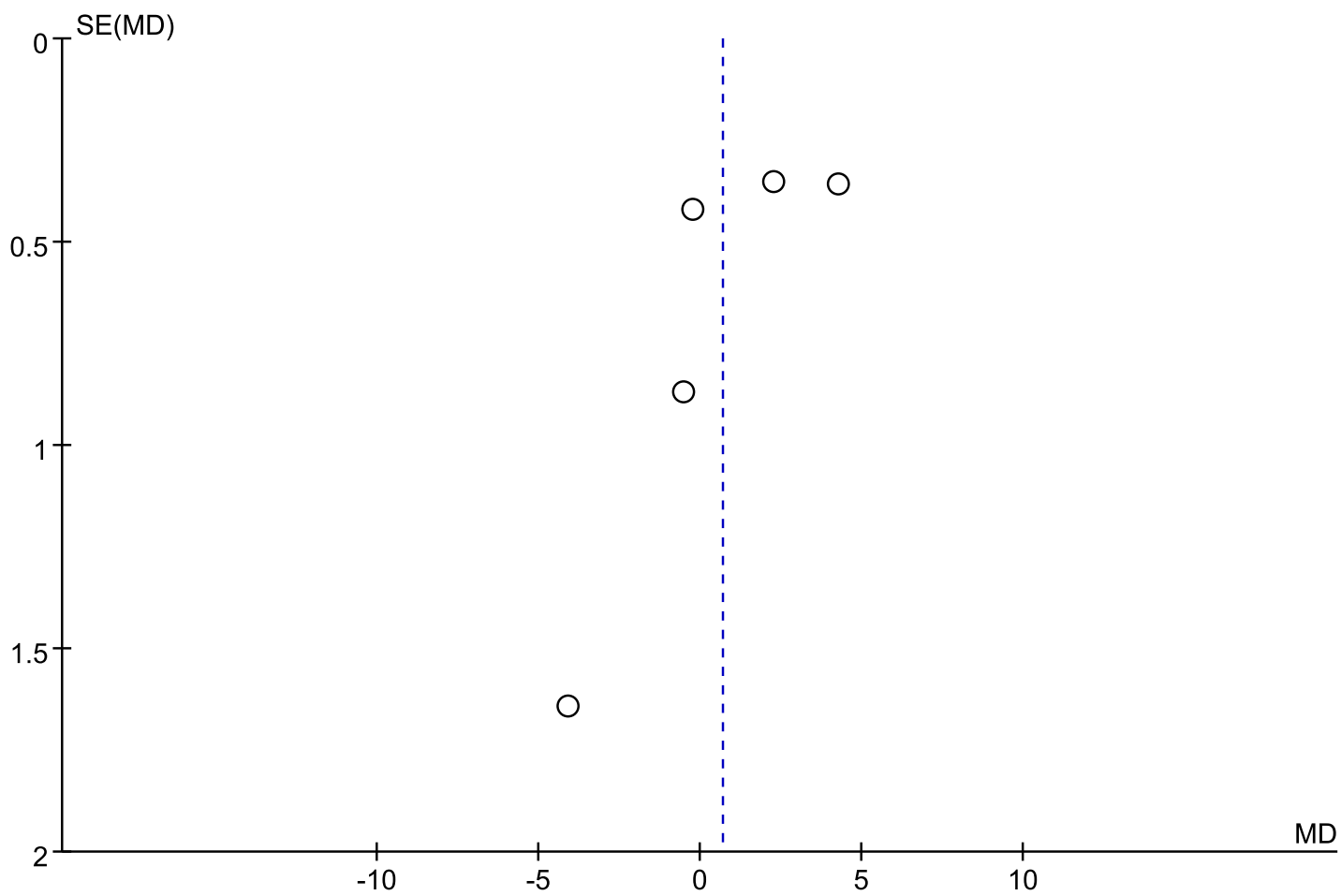

Supplement: Supplementary file 5 — Additional file 5: Figure S11a-Figure S11k. Funnel plot of FBG. Funnel plot of HbA1c. Funnel plot of FINS. Funnel plot of HOMA-IR. Funnel plot of TG. Funnel plot of TC. Funnel plot of HDL. Funnel plot of LDL. Funnel plot of SBP. Funnel plot of DBP. Funnel plot of BMI. [file 12986_2024_806_MOESM5_ESM.zip › Additional file 5/Figure S11c.pdf]

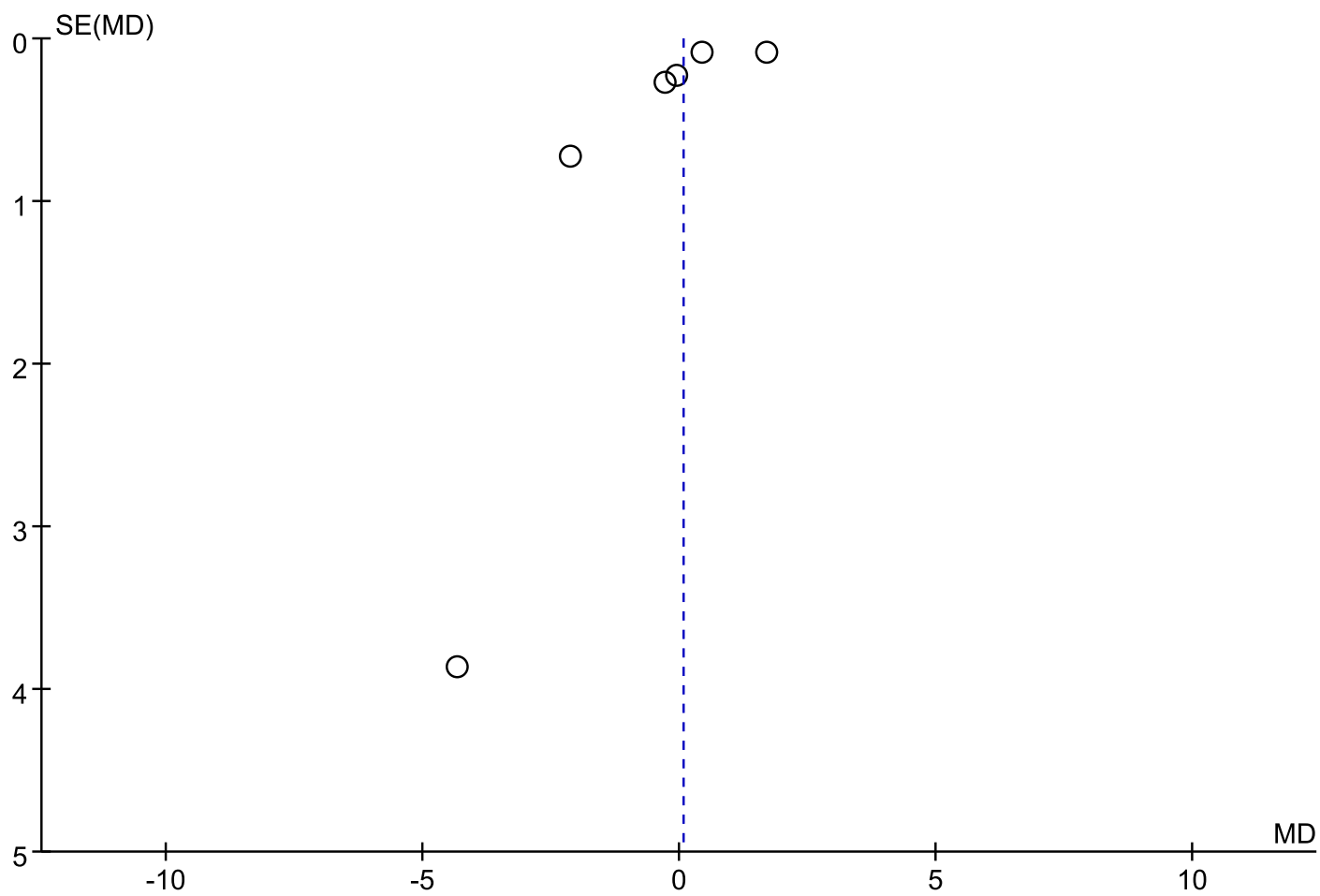

Supplement: Supplementary file 5 — Additional file 5: Figure S11a-Figure S11k. Funnel plot of FBG. Funnel plot of HbA1c. Funnel plot of FINS. Funnel plot of HOMA-IR. Funnel plot of TG. Funnel plot of TC. Funnel plot of HDL. Funnel plot of LDL. Funnel plot of SBP. Funnel plot of DBP. Funnel plot of BMI. [file 12986_2024_806_MOESM5_ESM.zip › Additional file 5/Figure S11d.pdf]

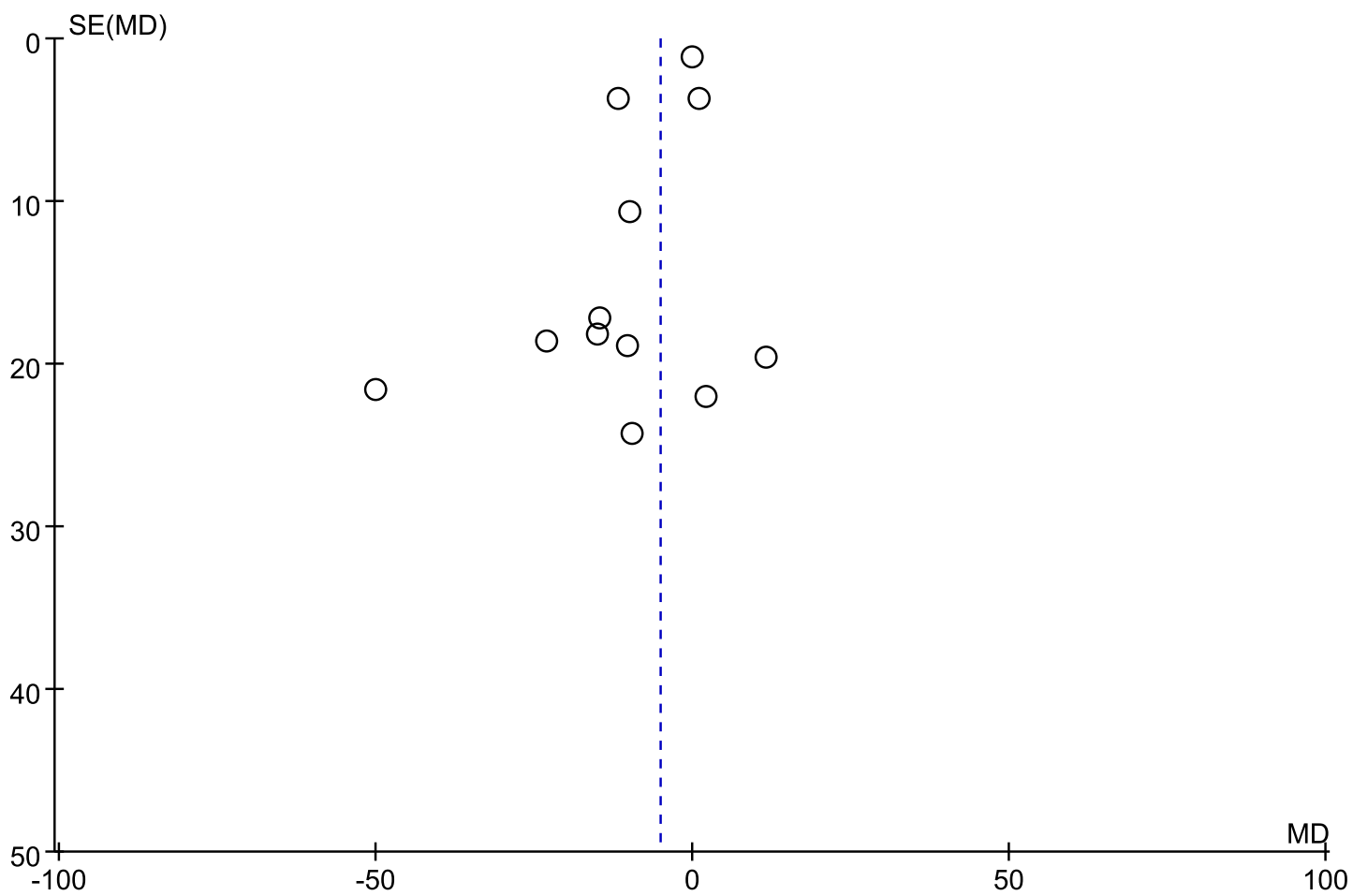

Supplement: Supplementary file 5 — Additional file 5: Figure S11a-Figure S11k. Funnel plot of FBG. Funnel plot of HbA1c. Funnel plot of FINS. Funnel plot of HOMA-IR. Funnel plot of TG. Funnel plot of TC. Funnel plot of HDL. Funnel plot of LDL. Funnel plot of SBP. Funnel plot of DBP. Funnel plot of BMI. [file 12986_2024_806_MOESM5_ESM.zip › Additional file 5/Figure S11e.pdf]

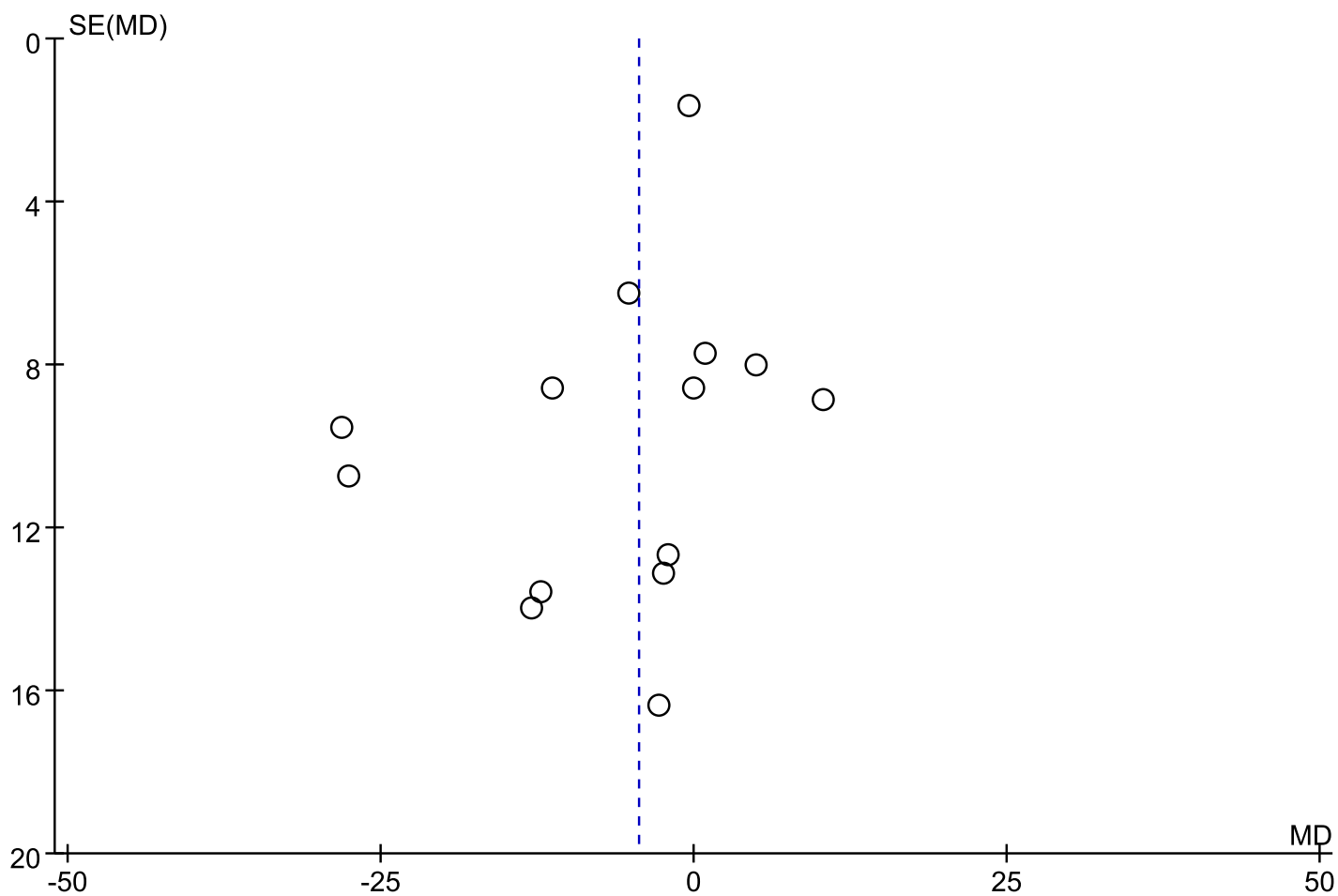

Supplement: Supplementary file 5 — Additional file 5: Figure S11a-Figure S11k. Funnel plot of FBG. Funnel plot of HbA1c. Funnel plot of FINS. Funnel plot of HOMA-IR. Funnel plot of TG. Funnel plot of TC. Funnel plot of HDL. Funnel plot of LDL. Funnel plot of SBP. Funnel plot of DBP. Funnel plot of BMI. [file 12986_2024_806_MOESM5_ESM.zip › Additional file 5/Figure S11f.pdf]

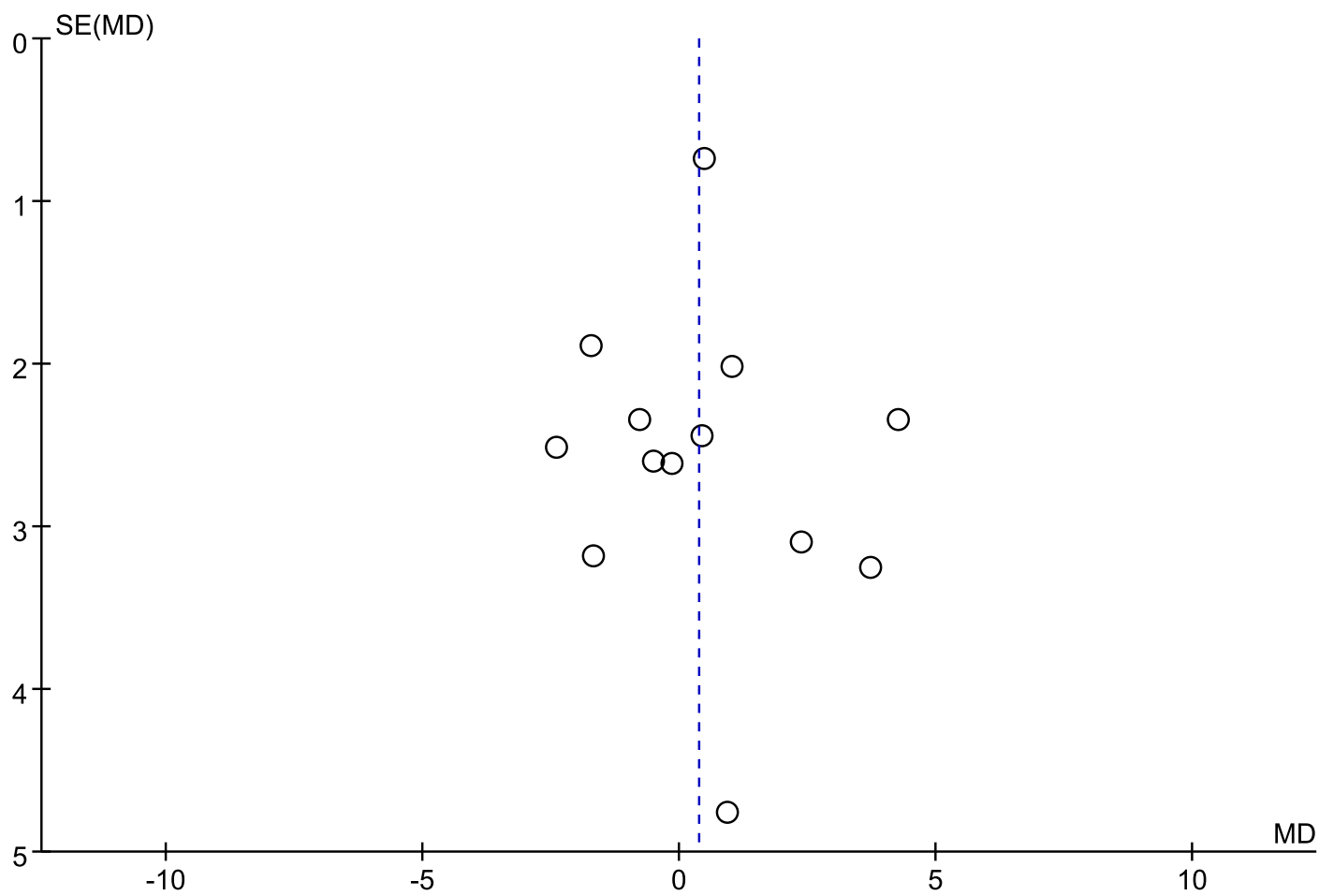

Supplement: Supplementary file 5 — Additional file 5: Figure S11a-Figure S11k. Funnel plot of FBG. Funnel plot of HbA1c. Funnel plot of FINS. Funnel plot of HOMA-IR. Funnel plot of TG. Funnel plot of TC. Funnel plot of HDL. Funnel plot of LDL. Funnel plot of SBP. Funnel plot of DBP. Funnel plot of BMI. [file 12986_2024_806_MOESM5_ESM.zip › Additional file 5/Figure S11g.pdf]

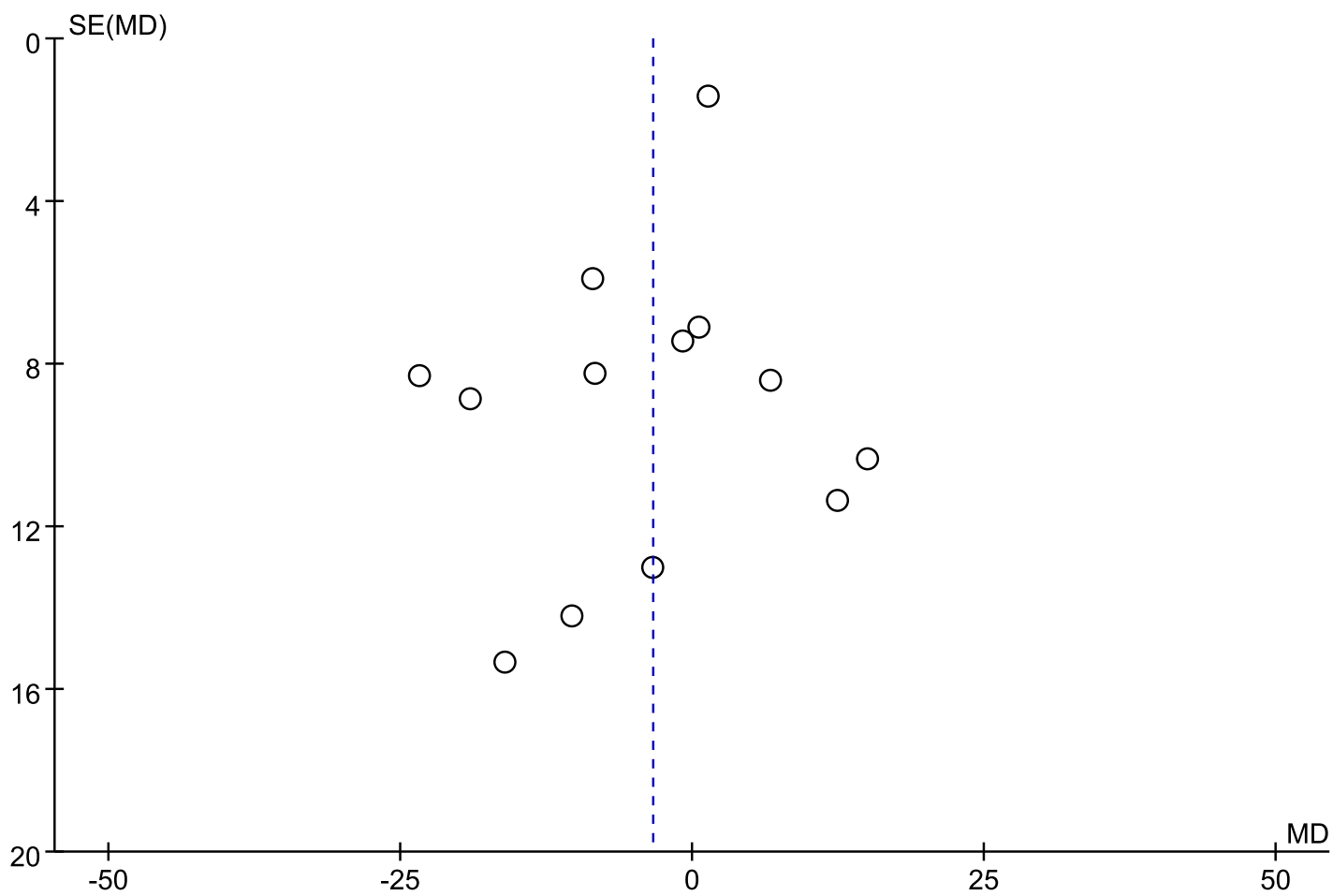

Supplement: Supplementary file 5 — Additional file 5: Figure S11a-Figure S11k. Funnel plot of FBG. Funnel plot of HbA1c. Funnel plot of FINS. Funnel plot of HOMA-IR. Funnel plot of TG. Funnel plot of TC. Funnel plot of HDL. Funnel plot of LDL. Funnel plot of SBP. Funnel plot of DBP. Funnel plot of BMI. [file 12986_2024_806_MOESM5_ESM.zip › Additional file 5/Figure S11h.pdf]

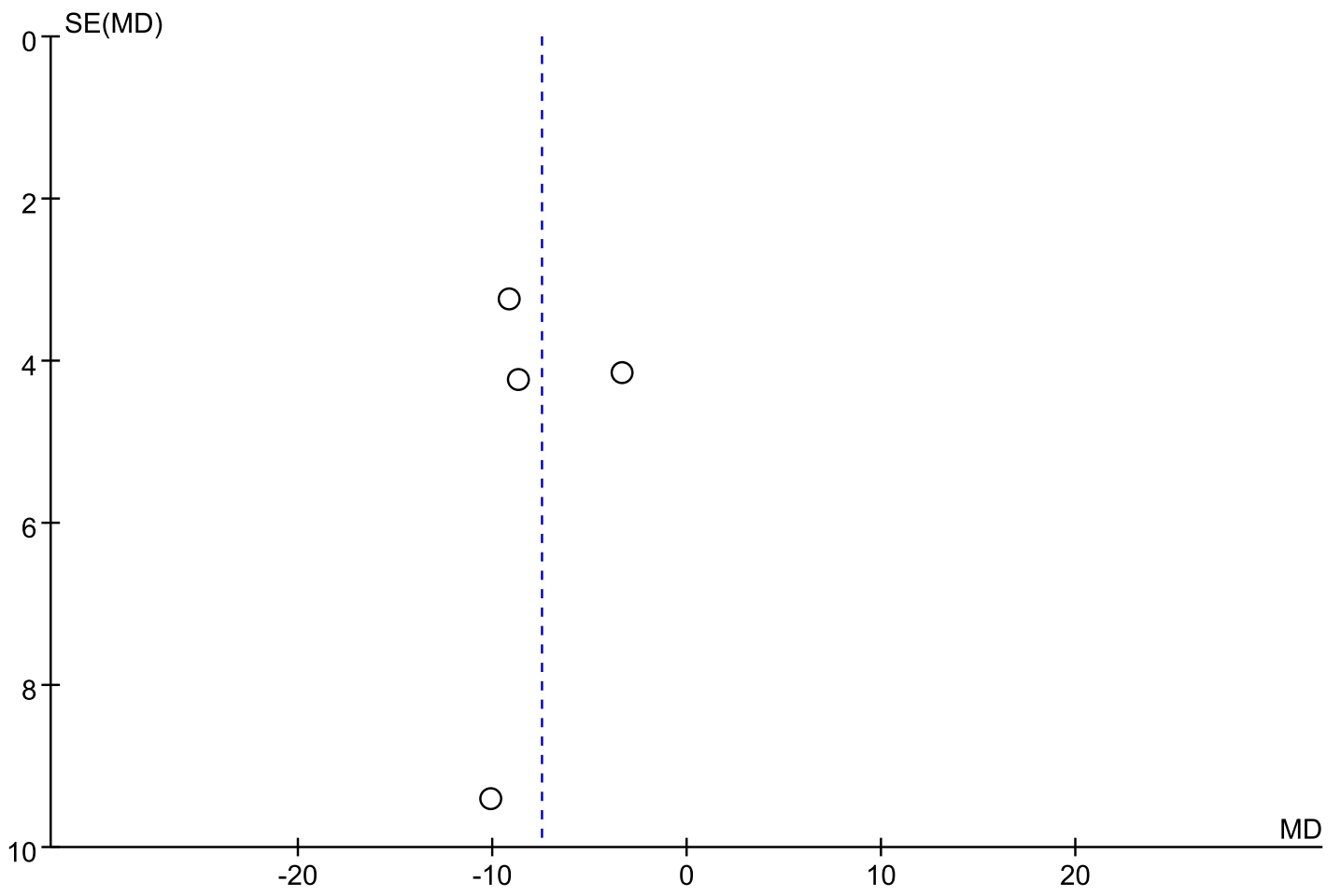

Supplement: Supplementary file 5 — Additional file 5: Figure S11a-Figure S11k. Funnel plot of FBG. Funnel plot of HbA1c. Funnel plot of FINS. Funnel plot of HOMA-IR. Funnel plot of TG. Funnel plot of TC. Funnel plot of HDL. Funnel plot of LDL. Funnel plot of SBP. Funnel plot of DBP. Funnel plot of BMI. [file 12986_2024_806_MOESM5_ESM.zip › Additional file 5/Figure S11i.pdf]

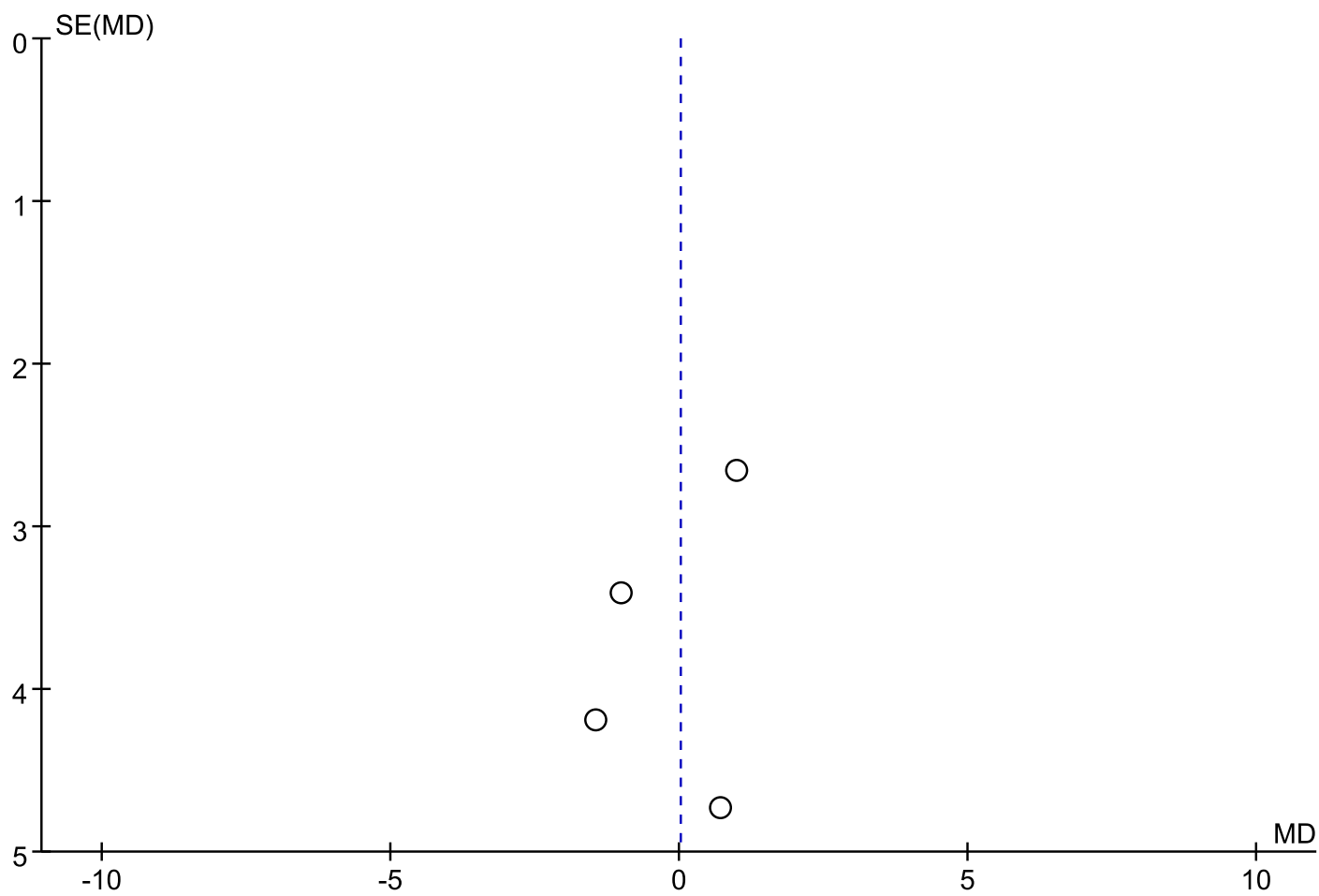

Supplement: Supplementary file 5 — Additional file 5: Figure S11a-Figure S11k. Funnel plot of FBG. Funnel plot of HbA1c. Funnel plot of FINS. Funnel plot of HOMA-IR. Funnel plot of TG. Funnel plot of TC. Funnel plot of HDL. Funnel plot of LDL. Funnel plot of SBP. Funnel plot of DBP. Funnel plot of BMI. [file 12986_2024_806_MOESM5_ESM.zip › Additional file 5/Figure S11j.pdf]

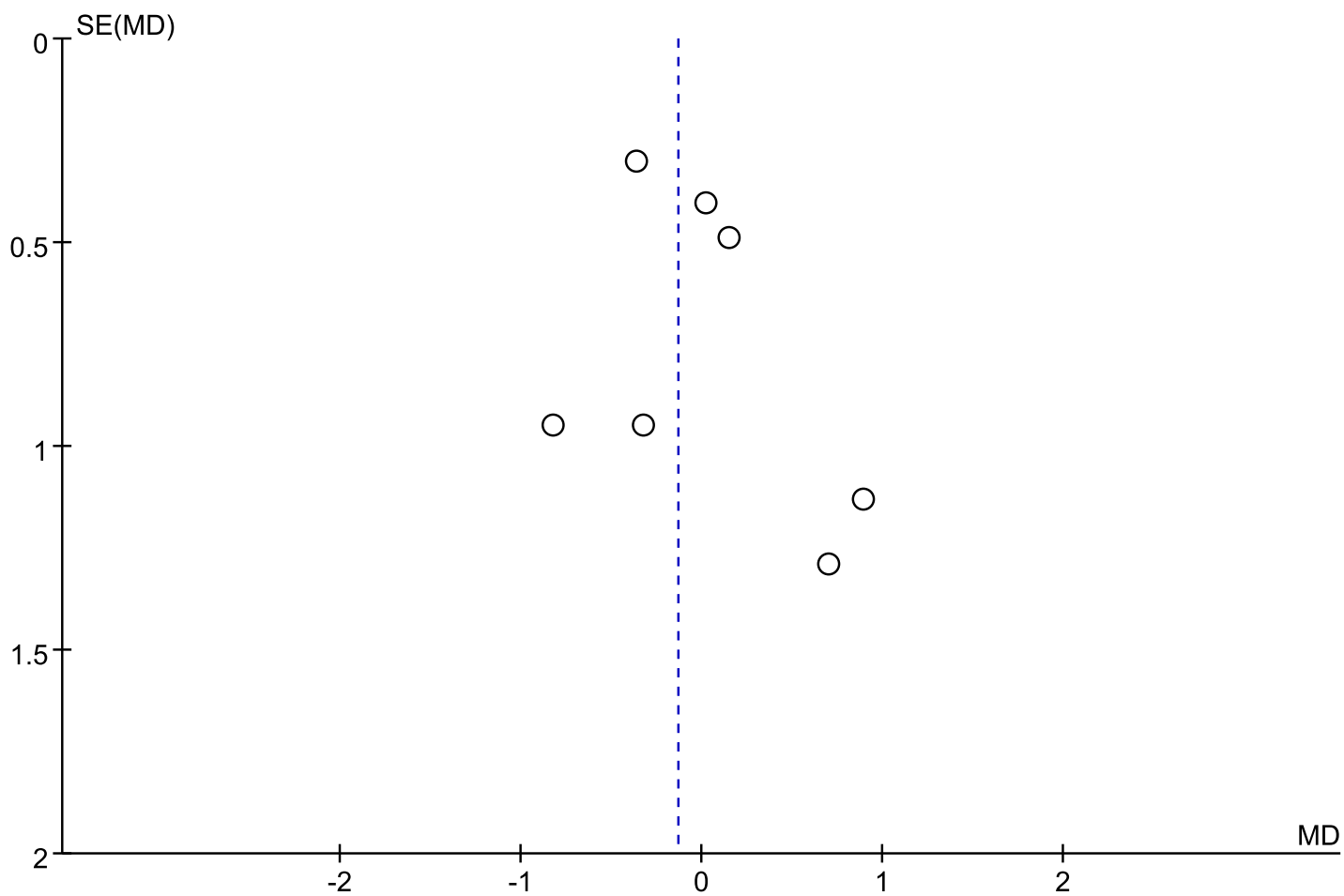

Supplement: Supplementary file 5 — Additional file 5: Figure S11a-Figure S11k. Funnel plot of FBG. Funnel plot of HbA1c. Funnel plot of FINS. Funnel plot of HOMA-IR. Funnel plot of TG. Funnel plot of TC. Funnel plot of HDL. Funnel plot of LDL. Funnel plot of SBP. Funnel plot of DBP. Funnel plot of BMI. [file 12986_2024_806_MOESM5_ESM.zip › Additional file 5/Figure S11k.pdf]

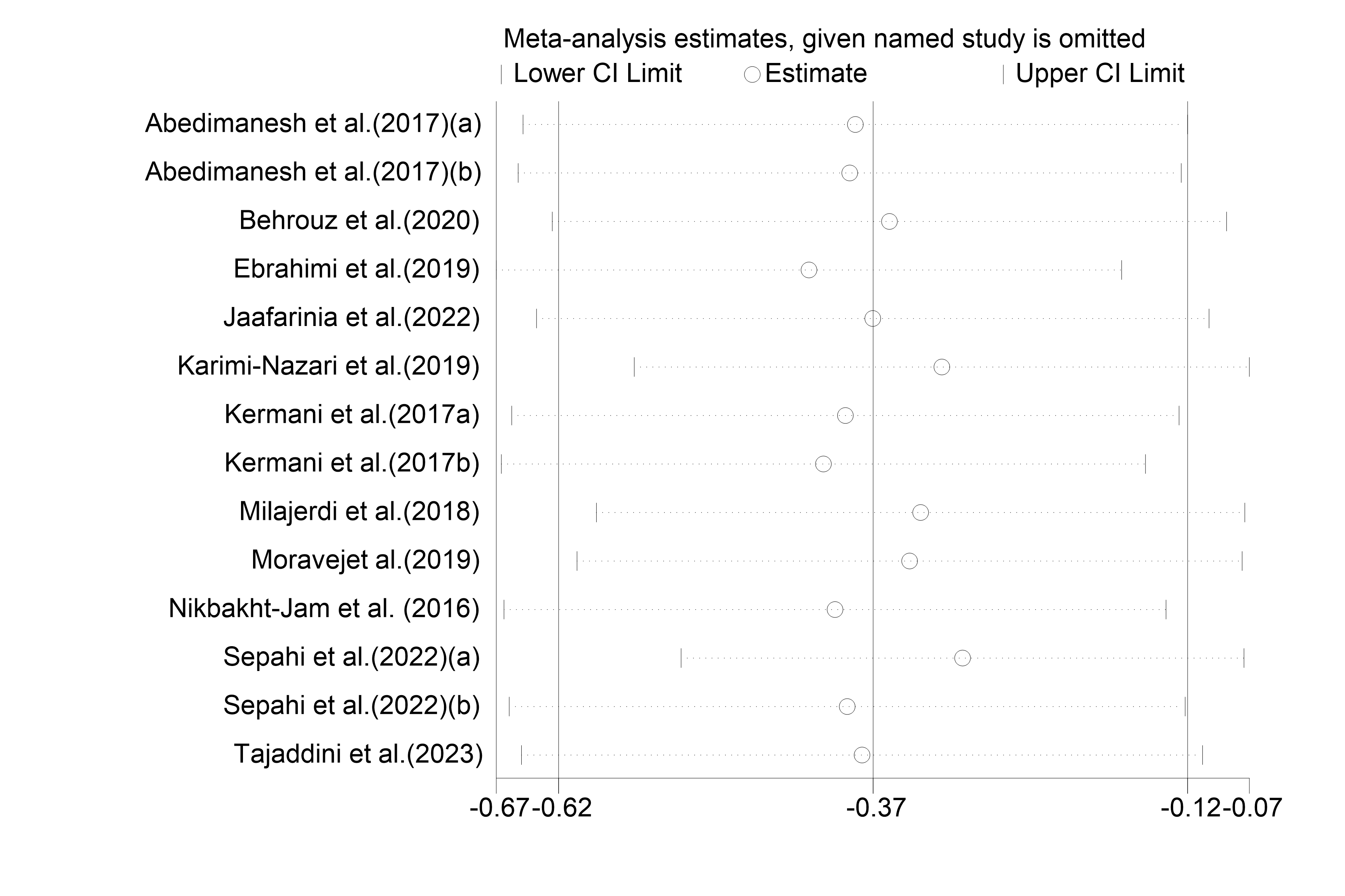

Supplement: Supplementary file 6 — Additional file 6: Figure S12a, Figure S12b, Figure S12c. Sensitivity analysis of FBG. Sensitivity analysis of HbA1c. Sensitivity analysis of SBP. [file 12986_2024_806_MOESM6_ESM.zip › Additional file 6/Fig.S12a.tif]

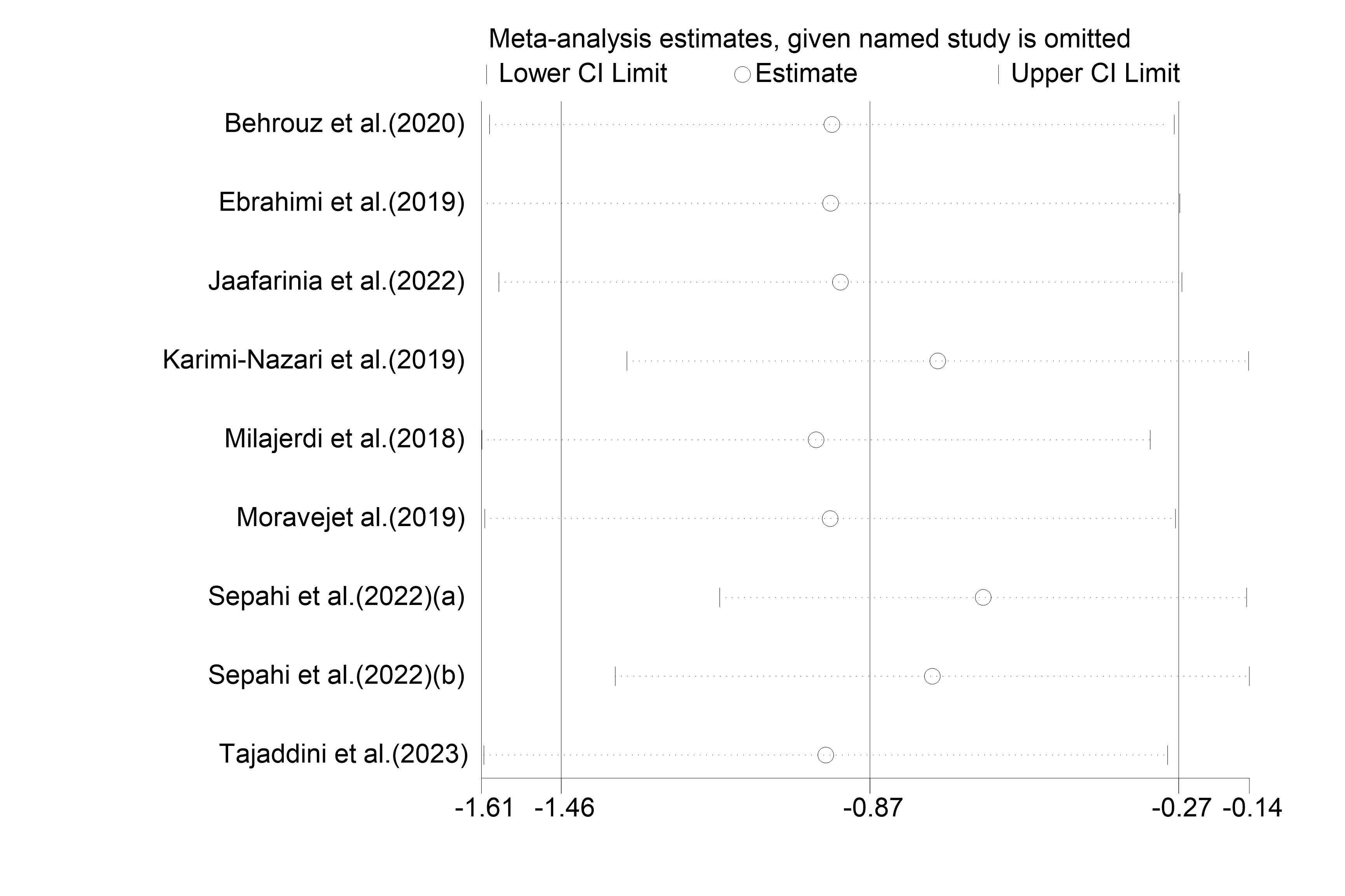

Supplement: Supplementary file 6 — Additional file 6: Figure S12a, Figure S12b, Figure S12c. Sensitivity analysis of FBG. Sensitivity analysis of HbA1c. Sensitivity analysis of SBP. [file 12986_2024_806_MOESM6_ESM.zip › Additional file 6/Fig.S12b.tif]

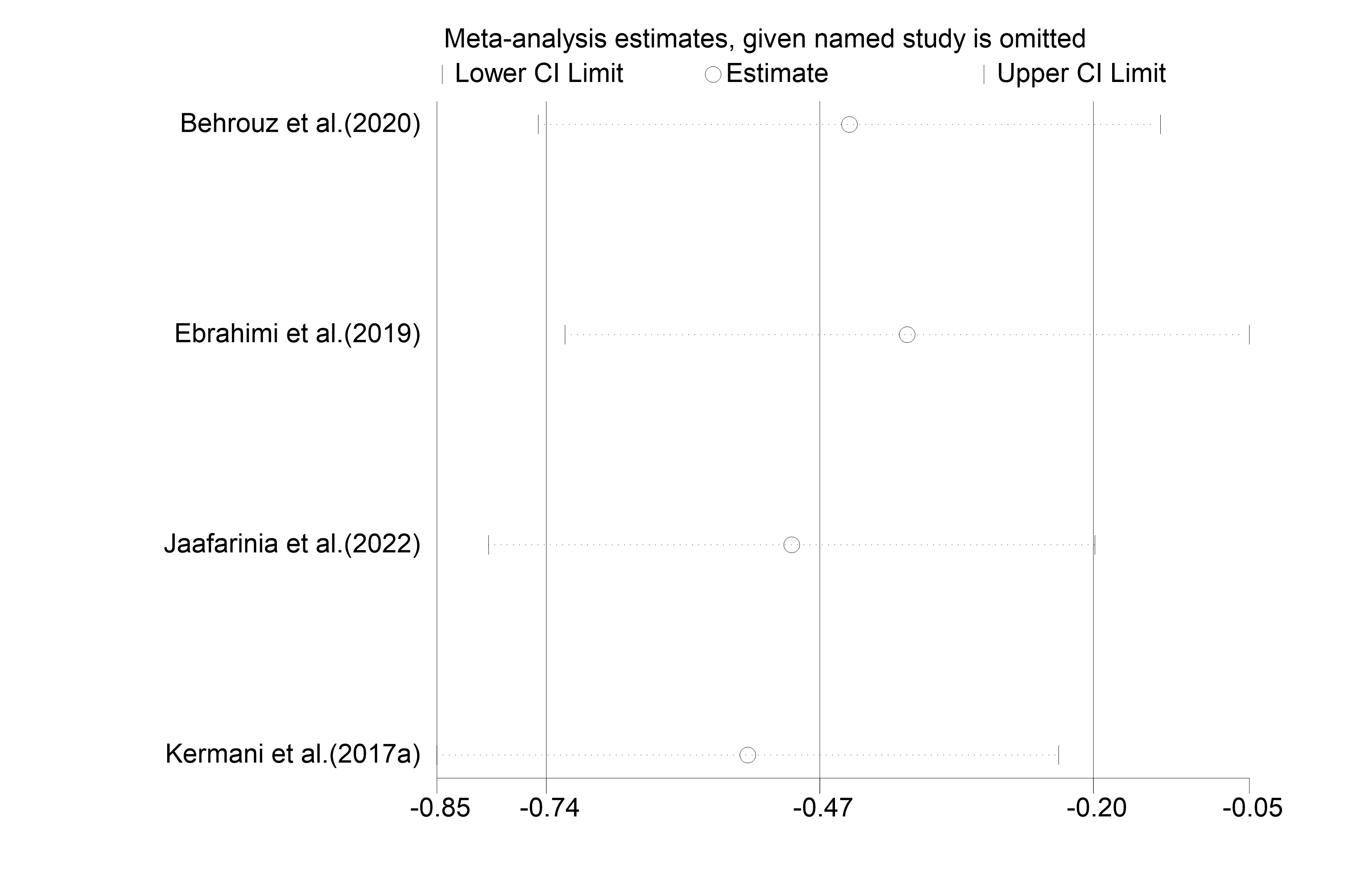

Supplement: Supplementary file 6 — Additional file 6: Figure S12a, Figure S12b, Figure S12c. Sensitivity analysis of FBG. Sensitivity analysis of HbA1c. Sensitivity analysis of SBP. [file 12986_2024_806_MOESM6_ESM.zip › Additional file 6/Fig.S12c.tif]
